# Development and validation of an interpretable machine learning model for concurrent identification of kinesiophobia in older patients with coronary heart disease after percutaneous coronary intervention

**DOI:** 10.3389/fmed.2026.1915056

**Published:** 2026-09-23

**Authors:** Yaya Xi, Meishan Zhang, Leyao Han, Minjie Liu, Jingying Xiong, Yunyun Liu, Xinman Dou, Bo Wan, Haixia Wang, Xinglei Wang

**Affiliations:** 1 School of Nursing, Lanzhou University, Lanzhou, China; 2 Department of Nursing, Lanzhou University Second Hospital, Lanzhou, China; 3 Department of Cardiovascular Medicine, Lanzhou University Second Hospital, Lanzhou, China; 4 The Sixth Ward of General Surgery, Lanzhou University Second Hospital, Lanzhou, Gansu, China

**Keywords:** coronary heart disease, kinesiophobia, machine learning, percutaneous coronary intervention, shap

## Abstract

**Background:**

Kinesiophobia reduces participation in cardiac rehabilitation and impairs exercise adherence among older patients with coronary heart disease (CHD) after percutaneous coronary intervention (PCI). This study aimed to develop and validate an interpretable machine learning (ML) model for identifying prevalent kinesiophobia in this population at the time of assessment.

**Methods:**

From December 2024 to August 2025, 502 older patients with CHD who underwent PCI were recruited from three tertiary grade-A hospitals in Lanzhou, Gansu Province, China. After stratification by kinesiophobia status, participants were randomly assigned to a development set (*n* = 352) and an internal validation set (*n* = 150). A further 150 patients recruited from the same hospitals between September and October 2025 constituted a temporal validation cohort. Five candidate algorithms were evaluated: Random Forest (RF), Logistic Regression (LR), Decision Tree (DT), eXtreme Gradient Boosting (XGBoost), and Support Vector Machine (SVM). Model development was conducted using a rigorous nested cross-validation framework with a 10-fold outer loop and a 5-fold inner loop. Data preprocessing, feature selection, synthetic minority oversampling technique (SMOTE) resampling, and hyperparameter tuning were independently repeated within the corresponding training partitions. The final algorithm was selected based on predictions generated from inner cross-validation within the development set, jointly considering discrimination, overall prediction error, calibration, and clinical net benefit.

**Results:**

A total of 15 predictors were identified, including the Barthel Index, avoidance, residence, fear of disease progression, clinical classification, confrontation, resignation, pain severity, educational level, smoking status, gender, depression, drinking status, exercise self-efficacy, and self-perceived burden. The mean outer-loop area under the receiver operating characteristic curve (AUC) values for LR, DT, RF, SVM, and XGBoost were 0.884 ± 0.076, 0.795 ± 0.069, 0.876 ± 0.071, 0.879 ± 0.072, and 0.878 ± 0.072, respectively. XGBoost demonstrated the best overall performance. The XGBoost model achieved an AUC of 0.821 [95% confidence interval (CI): 0.755–0.884] in the internal validation set and 0.981 (95% CI: 0.963–0.994) in the external validation set. The Brier scores were 0.170 and 0.062 in the internal and external validation sets, respectively. The corresponding calibration intercepts were 0.269 and 0.150, while the calibration slopes were 0.744 and 2.072, respectively.

**Conclusion:**

This study developed an interpretable ML model for the concurrent identification of kinesiophobia in older patients with CHD after PCI. After further validation in broader populations, the model may support early identification and personalized cardiac rehabilitation management.

## 1 Introduction

Coronary heart disease (CHD) is a prevalent cardiovascular disease and a major contributor to morbidity, mortality, and disease burden among older adults. As population ageing accelerates, the demand for long-term management of older patients with CHD continues to increase (1). Percutaneous coronary intervention (PCI) is currently a major treatment for CHD, as it can reopen narrowed or occluded coronary arteries, facilitate timely revascularization, and improve myocardial blood supply (2). Although PCI can effectively relieve coronary artery stenosis and myocardial ischemia, it does not halt the progression of underlying atherosclerosis. Consequently, patients remain at risk of restenosis, readmission, and adverse cardiovascular events after the procedure (3, 4).

Cardiac rehabilitation (CR) after PCI is therefore essential for improving long-term prognosis. CR is a comprehensive intervention that typically includes pharmacotherapy, exercise rehabilitation, nutritional guidance, psychological intervention, and health education. These components contribute to reduced cardiovascular mortality and readmission rates and improved quality of life (5). Among them, exercise rehabilitation is regarded as a core component of CR. However, participation in CR and adherence to exercise programmes remain suboptimal among older patients with CHD after PCI, with kinesiophobia representing an important psychological barrier (6). Kinesiophobia refers to an excessive fear of movement or exercise arising from concerns that physical activity may induce cardiac discomfort, aggravate disease status, or cause further injury (7). Previous studies have shown that kinesiophobia is common among patients with CHD and those undergoing PCI, with severe kinesiophobia reported more frequently in some older or hospitalized populations (8). Persistent fear of exercise not only weakens patients’ confidence in physical activity and reduces their participation in and adherence to CR but is also associated with negative emotions such as anxiety and depression, thereby further impairing functional recovery and long-term prognosis (9). Therefore, early identification of patients with kinesiophobia after PCI has important clinical value for providing targeted psychological support and exercise interventions (10).

At present, most studies on CHD have focused on identifying relevant risk factors and therapeutic targets in middle-aged patients, whereas older patients with CHD remain insufficiently investigated. Older adults constitute a key target population for exercise-based CR, as age-related declines in cardiopulmonary reserve, muscle strength, and physical function increase their susceptibility to sedentary behaviour and delayed functional recovery (11). Appropriately prescribed exercise-based CR may improve exercise tolerance and quality of life in older patients with CHD after PCI (12, 13). However, worries that physical activity may trigger cardiac discomfort or cardiovascular adverse events reduce patients’ willingness to join rehabilitation programmes, with kinesiophobia acting as a major barrier to sustained exercise adherence and functional recovery. Early screening of patients at high risk of kinesiophobia therefore enables timely psychological counselling, targeted exercise education, and tailored rehabilitation schemes. In recent years, machine learning (ML) models have been widely applied to clinical risk prediction owing to their ability to process high-dimensional data, capture complex nonlinear relationships, and support individualized risk stratification (14, 15). To our knowledge, no interpretable ML model has been developed specifically for the concurrent identification of kinesiophobia among older patients with CHD after PCI.

This study aimed to develop and validate an interpretable ML model based on multicenter data for the accurate identification of current kinesiophobia in older patients after PCI. In addition, the SHapley Additive exPlanations (SHAP) analysis was used to assess the relative contribution of each predictor and interpret individual level model outputs, thereby evaluating the model's clinical applicability for classifying kinesiophobia status and identifying patients with a high predicted probability of current kinesiophobia at the time of assessment.

## 2 Methods

### 2.1 Study population

This study employed convenience sampling to recruit older patients with CHD who underwent PCI in the cardiology departments of three tertiary grade-A hospitals in Lanzhou, Gansu Province, China, between December 2024 and August 2025. The sample size for model development was estimated using the formula for population prevalence in a sample survey: *N* = Z^2^ (*α*/2) × P(1-P)/*δ*^2^. With an allowable error of *δ* = 0.05, an expected prevalence of kinesiophobia of 76.0% among older patients with CHD (16), *α*=0.05, Z_0.05/2_=1.96, and *P*=0.76, the minimum required sample size was approximately 280. After accounting for a 20% rate of missing data, the required sample size was increased to 337. The target of 337 participants represented the minimum recruitment requirement. All eligible patients who provided written informed consent were consecutively enrolled until the end of the prespecified data-collection period, including after the minimum target sample size had been reached. The final sample size was determined by the number of eligible participants recruited during this period. A total of 510 patients were enrolled, of whom eight were excluded because their questionnaires did not meet the prespecified validity criteria. The final analytical sample comprised 502 participants, corresponding to a valid response rate of 98.4%. Before any missing-data imputation, data preprocessing, feature selection, resampling, or model fitting was performed, the participants were stratified by kinesiophobia status and randomly allocated to a development set (70%, *n* = 352) and an internal validation set (30%, *n* = 150). All model development procedures were conducted exclusively within the development set.

For external validation, older patients with CHD after PCI were prospectively recruited from the cardiology departments of the same three tertiary grade-A hospitals in Lanzhou, Gansu Province, between September and October 2025. The target sample size for external validation was set at approximately one-quarter to one-half of the initial internal cohort, with an additional 10% allowance for potential loss to follow-up or invalid data. Based on the initial cohort of 502 patients, the required sample size ranged from 139 to 279. A total of 157 patients were enrolled; after excluding invalid cases, 150 patients were included in the external validation cohort.

Inclusion criteria: ① confirmed diagnosis of CHD; ② undergone PCI; ③ aged ≥ 60 years; ④ consciousness with adequate verbal communication and comprehension abilities; and ⑤ voluntary participation in the study with written informed consent.

Exclusion criteria: ① confirmed diagnosis of a psychiatric disorder; ② severe underlying diseases, such as malignant tumours or leukaemia; ③ persistent severe arrhythmia or heart failure after PCI; ④ impaired limb mobility, such as hemiplegia or paralysis; and ⑤ severe organ dysfunction, such as liver enzyme levels ≥ 3 times the upper limit of normal or respiratory failure.

### 2.2 Study variables

#### 2.2.1 Predictor variables

A questionnaire assessing factors associated with kinesiophobia in older patients with CHD after PCI was developed based on literature review and expert meeting method. The questionnaire comprised 9 sections:

1. General demographic data: age, gender, marital status, educational level, residence, smoking status, and drinking status.
2. Disease-related information: body mass index (BMI), number of PCI procedures, procedure type, puncture site, physical activity level, number of comorbidities (e.g., hypertension, diabetes, hyperlipidemia), pain severity, clinical classification, time since PCI, and sedentary behaviour during the previous week.
3. Auxiliary examination indicators: laboratory and imaging data retrieved from hospital electronic medical records at admission, including total cholesterol (TC), left ventricular ejection fraction (LVEF), left ventricular end-diastolic volume (LVEDV), left ventricular end-systolic volume (LVESV), and left atrial diameter (LAD).
4. The Hospital Anxiety and Depression Scale (HADS), developed by Zigmond et al. (17), was used to assess anxiety and depression in hospitalized patients. The scale consists of two subscales for anxiety and depression, with 14 items in total, each scored on a 0–3 point scale, with higher scores indicating more severe symptoms of anxiety or depressive symptoms. In this study, the Cronbach's *α* coefficients for the anxiety and depression subscales were 0.813 and 0.799, respectively, indicating good internal consistency in the present sample.
5. The Medical Coping Modes Questionnaire (MCMQ), developed by Feifel H et al. (18), was used to assess coping strategies in clinical patients. The questionnaire comprises 20 items covering three dimensions: confrontation, avoidance, and resignation. Responses are rated on a 4-point Likert scale (1–4), including eight reverse-scored items. Subscale scores are calculated separately, with higher scores indicating a greater tendency to adopt the corresponding coping mode. In this study, the Cronbach's *α* coefficients for the confrontation, avoidance, and resignation subscales were 0.831, 0.732, and 0.803, respectively, indicating good internal consistency in the present sample.
6. The Self-Perceived Burden Scale (SPBS), developed by Cousineau (19). The scale consists of 10 items covering three dimensions: physical burden, emotional burden, and economic burden. It employs a 5-point Likert scale (1–5), with higher scores indicating greater self-perceived burden. In this study, the Cronbach's *α* coefficient for this scale was 0.883, indicating good internal consistency in the present sample.
7. The Self-Efficacy for Exercise (SEE) scale was developed by Resnick et al. (20). Comprising nine items, the scale assesses patients’ confidence in maintaining exercise under various conditions, such as adverse weather, pain, solitude, fatigue, and low mood. Each item is scored on a 0–10 scale, with higher scores indicating greater exercise self-efficacy. In this study, the scale's Cronbach's *α* coefficient was 0.953, indicating good internal consistency in the present sample.
8. The Barthel Index (BI), developed by Florence Mahoney and Dorothy Barthel (21), was used to assess activities of daily living. The index yields a total score ranging from 0 to 100, with higher scores indicating greater functional independence. Dependency categories were defined according to the prespecified score ranges. In this study, the Cronbach's *α* coefficient for the BI was 0.923, indicating good internal consistency in the present sample.
9. The Fear of Progression Questionnaire-Short Form (FoP-Q-SF), developed by Mehnert (22). It is a unidimensional simplified version consisting of 12 items. It employs a 5-point Likert scale, with higher scores indicating a more severe fear of disease progression. A score of 34 or above is used as the criterion for determining psychological dysfunction. In this study, the Cronbach's *α* for this scale was 0.890, indicating good internal consistency in the present sample.

#### 2.2.2 Outcome variable

This study used the Tampa Scale for Kinesiophobia Heart (TSK-SV Heart) to assess kinesiophobia among older patients with CHD after PCI. The TSK-SV Heart was adapted by Bäck et al. in Sweden from the original Tampa Scale for Kinesiophobia (10). The scale consists of 17 items across four dimensions: perceived danger, fear of exercise, exercise avoidance, and functional impairment. Total scores range from 17 to 68, with higher scores indicating greater exercise-related fear. In this study, a total score of ≥37 was used as the cut-off for identifying kinesiophobia. The Cronbach's *α* coefficient of the scale in this study was 0.904, indicating good internal consistency.

### 2.3 Feature selection within nested cross-validation

Feature selection was embedded within the cross-validation pipeline and independently repeated within each corresponding training partition. First, univariable logistic regression analyses were performed exclusively using data from the relevant training partition, and variables with an overall *P* < 0.05 were retained as candidate predictors. Least Absolute Shrinkage and Selection Operator (LASSO) logistic regression was subsequently applied to the variables retained through univariable screening. Within each training partition, five-fold cross-validation for a binomial outcome was used to determine lambda.min, defined as the parameter value corresponding to the minimum mean cross-validated binomial deviance. Variables with nonzero coefficients at lambda.min were retained. The Boruta algorithm was independently applied to the same set of candidate predictors within each training partition. Boruta assessed the importance of each original predictor by comparing it with randomly generated shadow variables. Predictors classified as “confirmed” or “tentative” were retained, whereas those classified as “rejected” were excluded. The intersection of the predictors retained by LASSO and Boruta constituted the feature set used for model fitting within the corresponding training partition.

### 2.4 Predictive model construction and evaluation

Five candidate ML algorithms were evaluated in this study, including Random Forest (RF), Logistic Regression (LR), Decision Tree (DT), eXtreme Gradient Boosting (XGBoost), and Support Vector Machine (SVM). Model development was performed using rigorous nested cross-validation, consisting of a 10-fold outer loop and a 5-fold inner loop. Both the outer and inner folds were stratified according to kinesiophobia status. The outer loop was used exclusively to estimate model performance within the development set. In each outer-loop iteration, the outer test fold remained completely independent of the model development process. Data preprocessing, feature selection, synthetic minority oversampling technique (SMOTE) resampling, model fitting, and hyperparameter tuning were performed using only the corresponding outer training data. Within each outer training fold, 5-fold inner cross-validation was conducted for hyperparameter tuning and comparison of the candidate algorithms. For each inner-loop split, missing-data imputation, categorical variable encoding, standardization, and feature selection were refitted solely based on the inner training subset. Following preprocessing and feature selection, SMOTE resampling was applied exclusively to the inner training subset. After the optimal hyperparameters were identified in the inner loop, the complete preprocessing and feature selection procedures were re-estimated using the corresponding outer training fold. SMOTE resampling was applied only to this outer training fold, and the fitted model was subsequently evaluated on the untouched outer test fold.

The candidate algorithms were compared using a prespecified, equally weighted rank-sum criterion based on predictions generated through inner cross-validation. Preference was given to a higher area under the receiver operating characteristic curve (AUC), a lower Brier score, a calibration intercept closer to 0, a calibration slope closer to 1, and a higher mean net benefit across threshold probabilities ranging from 0.10 to 0.50. The algorithm with the lowest overall rank sum was selected as the final algorithm. Neither the held-out internal validation cohort nor the external validation cohort was used for algorithm selection or hyperparameter tuning.

Model performance was evaluated in terms of discrimination, classification performance, overall probabilistic accuracy, calibration, and clinical utility. Discrimination was assessed using AUC. Classification performance was evaluated using accuracy, sensitivity, specificity, precision, recall, and the F1 score. Overall probabilistic accuracy was assessed using the Brier score. Calibration was evaluated using the calibration intercept, calibration slope, and calibration plots, whereas clinical utility was assessed using decision curve analysis.

Performance within the development set was calculated exclusively from out-of-fold predictions generated during the outer cross-validation loop and summarized as the mean ± standard deviation across the 10 outer folds. After the final model had been determined, its performance was independently evaluated in the held-out internal validation cohort and the external validation cohort. In the validation cohorts, confidence intervals for the AUC were estimated using the DeLong method, whereas confidence intervals for the remaining performance and calibration metrics were estimated using 1,000 bootstrap resamples. SHAP was used to interpret the final selected model, and global predictor importance was quantified using mean absolute SHAP values.

### 2.5 Statistical methods

Missing data handling was incorporated into the model development pipeline. During nested cross-validation, imputation rules were estimated exclusively from the corresponding training partition and subsequently applied to the relevant internal or external validation cohort. The distribution of each continuous variable was also assessed using only the corresponding training partition. Normally distributed continuous variables were imputed using the mean of the relevant training partition, non-normally distributed continuous variables were imputed using the median, and categorical variables were imputed using the mode. The proportion of missing data for each variable and the final imputation method are presented in Supplementary Tables S1–S4.

Descriptive statistics and between-group comparisons were performed using SPSS version 26.0 and R software. Normally distributed continuous variables are presented as the mean ± standard deviation, non-normally distributed continuous variables are presented as the median and interquartile range [M (P25, P75)], and categorical variables are presented as frequencies and percentages [*n* (%)]. For comparisons between participants with and without kinesiophobia, normally distributed continuous variables were analyzed using the independent-samples *t* test, and non-normally distributed continuous variables were analyzed using the Mann–Whitney U test. When comparing the development set, internal validation cohort, and external validation cohort, the distribution of each continuous variable was evaluated separately within each cohort. One-way analysis of variance was used only when the null hypothesis of normality was not rejected in all three cohorts; otherwise, the Kruskal–Wallis test was applied.

For categorical variables, Pearson's chi-square test was used when all expected cell counts were at least 1 and no more than 20% of the cells had expected counts below 5. Otherwise, an exact test was performed. When exact computation was infeasible because of the dimensionality or sparsity of the contingency table, *P* values were estimated using a Monte Carlo simulation with 10,000 replicates. All statistical tests were two-sided, and a *P* value < 0.05 was considered statistically significant.

## 3 Results

### 3.1 Baseline clinical information

A total of 502 older patients with CHD after PCI were included in this study, of whom 321 had kinesiophobia, corresponding to a prevalence of 63.9%. Among all enrolled patients, the median age was 63 years (interquartile range: 60–70), and the BMI was 24.44 ± 3.11 kg/m^2^. The cohort included 379 males (75.5%) and 123 females (24.5%). Regarding marital status, 468 patients (93.2%) were married, whereas 34 patients (6.8%) were single, widowed, or divorced. Educational level was distributed as follows: primary school or below, 33.3%; junior high school, 29.9%; senior high school or vocational school, 24.5%; and university or above, 12.3%. Rural and urban residents accounted for 40.8% and 59.2%, respectively. The proportions of patients who smoked and consumed alcohol were 37.3% and 24.1%, respectively. Statistically significant differences between participants with and without kinesiophobia were observed in gender, educational level, residence, physical activity, sedentary behaviour, drinking status, smoking, confrontation, avoidance, resignation, exercise self-efficacy, number of PCI procedures, procedure type, pain severity, clinical classification, anxiety, depression, self-perceived burden, Barthel Index, and fear of disease progression. Detailed results are presented in Table 1. After stratification according to kinesiophobia status, the initial internal cohort was randomly divided into a development set and a holdout internal validation set at a ratio of 7:3, comprising 352 and 150 participants, respectively. Kinesiophobia was present in 225 participants (63.9%) in the development set and 96 participants (64.0%) in the internal validation set. The external validation set included 150 participants, of whom 88 (58.7%) had kinesiophobia. There was no statistically significant difference in the prevalence of kinesiophobia among the three cohorts (*P* = 0.503). Characteristics of the external validation set stratified by kinesiophobia status are presented in Supplementary Table 5, comparisons of baseline characteristics among the development set, internal validation set, and external validation set are presented in Supplementary Table 6, and the study flowchart is shown in Supplementary Figure 1.

**Table 1:** Baseline characteristics of patients.

Variables	Total (*n* = 502)	Kinesiophobia group (*n* = 321)	Non-kinesiophobia group (*n* = 181)	Statistical value	*P*
Age, Median (IQR)	63 (60, 70)	63 (60, 71)	64 (60, 70)	−0.534^a^	0.593
BMI, (Mean ± SD)	24.44 ± 3.113	24.40 ± 3.105	24.53 ± 3.134	−0.460^b^	0.646
Gender, *n* (%)				15.725^c^	< 0.001
Male	379 (75.5)	224 (59.1)	155 (40.9)		
Female	123 (24.5)	97 (78.9)	26 (21.1)		
Marital status, *n* (%)				–	0.937^d^
Married	468 (93.2)	298 (63.7)	170 (36.3)		
Unmarried	3 (0.6)	2 (66.7)	1 (33.3)		
Widowed	29 (5.8)	20 (69.0)	9 (31.0)		
Divorced	2 (0.4)	1 (50.0)	1 (50.0)		
Educational level, *n* (%)				8.679^c^	0.034
Primary school or below	167 (33.3)	116 (69.5)	51 (30.5)		
Junior high school	150 (29.9)	100 (66.7)	50 (33.3)		
Senior high school/technical secondary school	123 (24.5)	74 (60.2)	49 (39.8)		
College or above	62 (12.3)	31 (50.0)	31 (50.0)		
Residence, *n* (%)				5.076^c^	0.024
Rural	205 (40.8)	143 (69.8)	62 (30.2)		
Urban	297 (59.2)	178 (59.9)	119 (40.1)		
Physical activity level, *n* (%)				20.120^c^	< 0.001
Low	269 (53.6)	196 (72.9)	73 (27.1)		
Moderate	192 (38.2)	104 (54.2)	88 (45.8)		
High	41 (8.2)	21 (51.2)	20 (48.8)		
Sedentary behaviour, *n* (%)				16.503^c^	< 0.001
No	239 (47.6)	131 (54.8)	108 (45.2)		
Yes	263 (52.4)	190 (72.2)	73 (27.8)		
Drinking status, *n* (%)				8.808^c^	0.012
Never drinker	255 (50.8)	179 (70.2)	76 (29.8)		
Former drinker	126 (25.1)	73 (57.9)	53 (42.1)		
Current drinker	121 (24.1)	69 (57.0)	52 (43.0)		
Smoking status, *n* (%)				11.002^c^	0.004
Never smoker	197 (39.2)	143 (72.6)	54 (27.4)		
Former smoker	118 (23.5)	66 (55.9)	52 (44.1)		
Current smoker	187 (37.3)	112 (59.9)	75 (40.1)		
Time since PCI (days), Median (IQR)	1 (1, 1)	1 (1, 1)	1 (1, 1)	−0.942^a^	0.346
TC (mmol/L), Median (IQR)	3.54 (2.97, 4.44)	3.58 (3.04, 4.56)	3.42 (2.84, 4.28)	−1.381^a^	0.167
LVEDV (ml), Median (IQR)	100.00 (84.00, 116.25)	100.0 (84.0, 117.5)	98.0 (83.0, 116.0)	−0.946^a^	0.344
LVESV (ml), Median (IQR)	37.00 (29.00, 48.00)	37.0 (29.0, 50.0)	36.0 (29.0, 44.5)	−1.350^a^	0.177
LAD (mm), Median (IQR)	34.00 (31.00, 37.00)	34.0 (31.0, 37.5)	34.0 (31.0, 37.0)	−0.871^a^	0.384
Medical Coping Modes, Median (IQR)					
Confrontation	17 (13, 19)	16 (13, 19)	18 (15, 21)	−4.679^a^	< 0.001
Avoidance	16 (13, 18)	17 (15, 19)	14 (12, 16)	−7.868^a^	< 0.001
Resignation	7 (5, 10)	9 (6, 11)	5 (5, 7)	−9.633^a^	< 0.001
Exercise self-efficacy, Median (IQR)	5.22 (3.08, 8.00)	4.11 (2.33, 6.17)	7.78 (5.22, 8.83)	−10.360^a^	< 0.001
Number of PCI procedures, *n* (%)				7.445^c^	0.024
1	301 (60.0)	206 (68.4)	95 (31.6)		
2	160 (31.9)	89 (55.6)	71 (44.4)		
≥3	41 (8.1)	26 (63.4)	15 (36.6)		
Procedure type, *n* (%)				3.997^c^	0.046
Elective	471 (93.8)	296 (62.8)	175 (37.2)		
Emergency	31 (6.2)	25 (80.6)	6 (19.4)		
Puncture site, *n* (%)				1.354^c^	0.244
Radial artery	459 (91.4)	290 (63.2)	169 (36.8)		
Femoral artery	43 (8.6)	31 (72.1)	12 (27.9)		
Number of comorbidities, *n* (%)				1.626^c^	0.654
0	130 (25.9)	82 (63.1)	48 (36.9)		
1	207 (41.2)	134 (64.7)	73 (35.3)		
2	117 (23.3)	71 (60.7)	46 (39.3)		
≥3	48 (9.6)	34 (70.8)	14 (29.2)		
Pain severity, *n* (%)				–	< 0.001^d^
Mild pain	436 (86.8)	265 (60.8)	171 (39.2)		
Moderate pain	56 (11.2)	46 (82.1)	10 (17.9)		
Severe pain	10 (2.0)	10 (100.0)	0 (0.0)		
Clinical classification, *n* (%)				5.677^c^	0.017
CCS	77 (15.3)	40 (51.9)	37 (48.1)		
ACS	425 (84.7)	281 (66.1)	144 (33.9)		
LVEF (%), *n* (%)				–	0.106^d^
<40	20 (4.0)	16 (80.0)	4 (20.0)		
40∼49	36 (7.2)	27 (75.0)	9 (25.0)		
≥50	446 (88.8)	278 (62.3)	168 (37.7)		
Anxiety, *n* (%)				–	< 0.001^d^
No anxiety	367 (73.1)	196 (53.4)	171 (46.6)		
Possible anxiety	74 (14.7)	68 (91.9)	6 (8.1)		
Anxiety	61 (12.2)	57 (93.4)	4 (6.6)		
Depression, *n* (%)				–	< 0.001^d^
No depression	331 (65.9)	161 (48.6)	170 (51.4)		
Possible depression	93 (18.5)	86 (92.5)	7 (7.5)		
Depression	78 (15.6)	74 (94.9)	4 (5.1)		
Self-perceived burden, *n* (%)				–	< 0.001^d^
No obvious burden	251 (50.0)	115 (45.8)	136 (54.2)		
Mild burden	173 (34.5)	132 (76.3)	41 (23.7)		
Moderate burden	70 (13.9)	66 (94.3)	4 (5.7)		
Severe burden	8 (1.6)	8 (100.0)	0 (0.0)		
Barthel Index, *n* (%)				–	< 0.001^d^
Independent	405 (80.7)	240 (59.3)	165 (40.7)		
Mild dependence	74 (14.7)	63 (85.1)	11 (14.9)		
Moderate dependence	18 (3.6)	14 (77.8)	4 (22.2)		
Severe dependence	5 (1.0)	4 (80.0)	1 (20.0)		
Fear of disease progression, *n* (%)				66.963^c^	< 0.001
Present	125 (24.9)	118 (94.4)	7 (5.6)		
Absent	377 (75.1)	203 (53.8)	174 (46.2)		

BMI, body mass index; TC, total cholesterol; LVEF, left ventricular ejection fraction; LVEDV, left ventricular end-diastolic volume; LVESV, left ventricular end-systolic volume; LAD, left atrial diameter; ACS, acute coronary syndrome; CCS, chronic coronary syndrome; IQR, interquartile range.

Percentages in the kinesiophobia and non-kinesiophobia group columns are row percentages.

– no value available.

a *Z* value in the Mann–Whitney U-test.

b *t* value in a *t*-test.

c *χ^2^* value in Chi-square test.

d *p* value in Fisher's exact test.

### 3.2 Feature selection

Univariable logistic regression identified 19 candidate variables. Among these candidates, least absolute shrinkage and selection operator (LASSO) regression with 5-fold cross-validation was performed, yielding an optimal penalty parameter of lambda.min = 0.013809477. At this value of lambda.min, 16 variables had nonzero coefficients: Barthel Index, number of PCI procedures, avoidance, residence, fear of disease progression, clinical classification, confrontation, resignation, pain severity, educational level, smoking status, gender, depression, drinking status, exercise self-efficacy, and self-perceived burden.

The Boruta algorithm retained 16 variables classified as either Confirmed or Tentative: gender, educational level, residence, pain severity, clinical classification, drinking status, smoking status, anxiety, depression, confrontation, avoidance, resignation, self-perceived burden, exercise self-efficacy, Barthel Index, and fear of disease progression.

The intersection of the variables selected by LASSO and Boruta comprised 15 variables: Barthel Index, avoidance, residence, fear of disease progression, clinical classification, confrontation, resignation, pain severity, educational level, smoking status, gender, depression, drinking status, exercise self-efficacy, and self-perceived burden. These 15 variables constituted the locked feature set used for the final model refitting on the full development set. The corresponding feature-selection results are shown in Figure 1.

**Figure 1:**
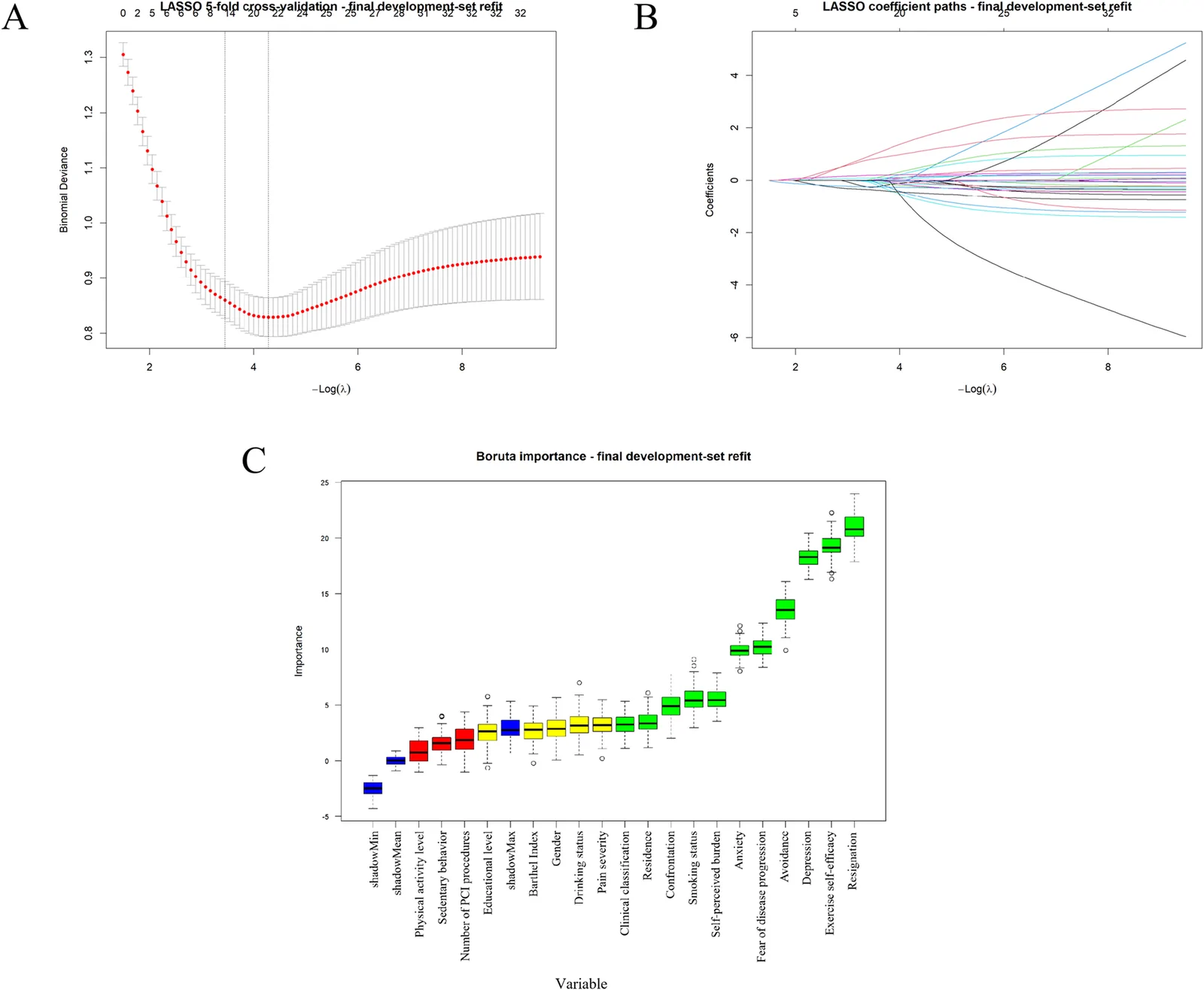
Feature selection using LASSO and Boruta. **(A)** Five-fold cross-validation curve for the LASSO logistic regression during the final refitting of the complete development set. The value of *λ* minimizing the cross-validated binomial deviance was selected (lambda.min = 0.013809477), retaining 16 candidate variables. **(B)** LASSO coefficient paths showing coefficient shrinkage with increasing regularization. **(C)** Boruta feature-importance analysis conducted during the final development-set refit. Sixteen variables classified as Confirmed or Tentative were retained.

### 3.3 Nested cross-validation and internal validation

Five candidate algorithms, including LR, DT, RF, SVM, and XGBoost, were compared using rigorous nested cross-validation. All performance metrics for the development set were derived from out-of-fold predictions generated from the outer test folds. The mean outer-fold AUC values for LR, DT, RF, SVM, and XGBoost were 0.884 ± 0.076, 0.795 ± 0.069, 0.876 ± 0.071, 0.879 ± 0.072, and 0.878 ± 0.072, respectively. Additional evaluation metrics, including accuracy, sensitivity, specificity, Brier score, and calibration intercept, are presented in Table 2.

**Table 2:** Comparative performance of five models in the development set using nested cross-validation.

Model	AUC	Accuracy	F1	Precision	Recall	Sensitivity	Specificity	Brier score	Calibration intercept	Calibration slope
DT	0.795 ± 0.069	0.770 ± 0.068	0.808 ± 0.059	0.861 ± 0.067	0.769 ± 0.092	0.769 ± 0.092	0.772 ± 0.134	0.178 ± 0.051	0.593 ± 0.482	0.671 ± 0.229
LR	0.884 ± 0.076	0.804 ± 0.085	0.835 ± 0.074	0.896 ± 0.075	0.786 ± 0.093	0.786 ± 0.093	0.837 ± 0.117	0.138 ± 0.054	0.599 ± 0.381	0.925 ± 0.669
RF	0.876 ± 0.071	0.796 ± 0.078	0.833 ± 0.068	0.864 ± 0.062	0.808 ± 0.099	0.808 ± 0.099	0.774 ± 0.109	0.138 ± 0.038	0.322 ± 0.354	1.260 ± 0.535
SVM	0.879 ± 0.072	0.807 ± 0.081	0.837 ± 0.071	0.896 ± 0.063	0.791 ± 0.102	0.791 ± 0.102	0.836 ± 0.110	0.140 ± 0.048	0.520 ± 0.373	1.020 ± 0.604
XGBoost	0.878 ± 0.072	0.800 ± 0.063	0.834 ± 0.056	0.874 ± 0.057	0.804 ± 0.099	0.804 ± 0.099	0.788 ± 0.104	0.140 ± 0.044	0.435 ± 0.410	1.348 ± 1.427

Values are presented as mean ± standard deviation across the 10 outer test folds of nested cross-validation.

LR, logistic regression; DT, decision tree; RF, random forest; XGBoost, eXtreme gradient boosting; SVM, support vector machine; AUC, area under receiver operating characteristic curve; F1, F1 score.

The final algorithm was selected exclusively on the basis of a prespecified multi-metric composite ranking derived from predictions generated during inner cross-validation within the development set. The ranking criteria favored a higher AUC, a lower Brier score, a calibration intercept closer to 0, a calibration slope closer to 1, and a higher mean net benefit across threshold probabilities ranging from 0.10 to 0.50. Based on this composite ranking, XGBoost achieved the best overall rank and was selected as the final algorithm before internal and external validation were conducted, as shown in Supplementary Table 7. Neither the internal validation set nor the external validation set was involved in algorithm selection or hyperparameter tuning.

In the internal validation set, XGBoost achieved an AUC of 0.821 (95% CI, 0.755–0.884), with an accuracy of 0.760, sensitivity of 0.781, specificity of 0.722, precision of 0.833, and an F1 score of 0.806. The Brier score was 0.170 (95% bootstrap CI, 0.134–0.209), the calibration intercept was 0.269 (95% bootstrap CI, −0.195–0.777), and the calibration slope was 0.744 (95% bootstrap CI, 0.527–1.055), as presented in Table 3. The receiver operating characteristic curve, calibration curve, and decision curve for the internal validation set are shown in Figures 2A–C.

**Table 3:** Performance of five models in internal and external validation.

Model	AUC	Accuracy	F1	Precision	Recall	Sensitivity	Specificity	Brier score	Calibration slope	Calibration intercept
Internal validation set										
RF	0.800 (0.728–0.865)	0.727 (0.653–0.787)	0.794 (0.725–0.848)	0.767 (0.689–0.849)	0.823 (0.740–0.896)	0.823 (0.740–0.896)	0.555 (0.424–0.682)	0.175 (0.142–0.211)	0.770 (0.523–1.143)	0.061 (−0.351–0.528)
LR	0.785 (0.710–0.852)	0.720 (0.647–0.787)	0.764 (0.687–0.830)	0.829 (0.744–0.904)	0.708 (0.613–0.800)	0.708 (0.613–0.800)	0.741 (0.625–0.850)	0.212 (0.164–0.263)	0.359 (0.245–0.524)	0.447 (−0.264–1.175)
DT	0.743 (0.671–0.816)	0.720 (0.647–0.787)	0.777 (0.711–0.839)	0.793 (0.714–0.872)	0.760 (0.680–0.842)	0.760 (0.680–0.842)	0.648 (0.518–0.771)	0.217 (0.169–0.264)	0.491 (0.317–0.702)	0.556 (−0.051–1.193)
XGBoost	0.821 (0.755–0.884)	0.760 (0.693–0.820)	0.806 (0.742–0.863)	0.833 (0.761–0.903)	0.781 (0.691–0.857)	0.781 (0.691–0.857)	0.722 (0.606–0.830)	0.170 (0.134–0.209)	0.744 (0.527–1.055)	0.269 (−0.195–0.777)
SVM	0.807 (0.733–0.870)	0.733 (0.660–0.807)	0.780 (0.707–0.841)	0.826 (0.745–0.899)	0.740 (0.648–0.826)	0.740 (0.648–0.826)	0.722 (0.604–0.831)	0.189 (0.146–0.235)	0.523 (0.361–0.760)	0.426 (−0.139–1.030)
External validation set										
RF	0.977 (0.956–0.992)	0.907 (0.860–0.947)	0.920 (0.872–0.957)	0.920 (0.863–0.967)	0.920 (0.857–0.968)	0.920 (0.857–0.968)	0.887 (0.810–0.954)	0.074 (0.057–0.093)	2.523 (1.946–3.868)	−0.045 (−0.377–0.252)
LR	0.978 (0.958–0.993)	0.913 (0.867–0.953)	0.923 (0.875–0.961)	0.963 (0.918–1.000)	0.886 (0.812–0.949)	0.886 (0.812–0.949)	0.952 (0.894–1.000)	0.063 (0.039–0.091)	1.269 (0.951–2.024)	0.541 (−0.006–1.029)
DT	0.882 (0.821–0.935)	0.860 (0.800–0.907)	0.883 (0.825–0.924)	0.868 (0.795–0.928)	0.898 (0.826–0.957)	0.898 (0.826–0.957)	0.806 (0.700–0.893)	0.118 (0.083–0.159)	1.040 (0.814–1.378)	0.126 (−0.297–0.567)
XGBoost	0.981 (0.963–0.994)	0.920 (0.873–0.960)	0.930 (0.885–0.968)	0.952 (0.905–0.988)	0.909 (0.840–0.967)	0.909 (0.840–0.967)	0.935 (0.873–0.984)	0.062 (0.043–0.084)	2.072 (1.633–3.328)	0.150 (−0.210–0.473)
SVM	0.985 (0.968–0.995)	0.913 (0.867–0.953)	0.922 (0.875–0.961)	0.975 (0.936–1.000)	0.875 (0.800–0.943)	0.875 (0.800–0.943)	0.967 (0.917–1.000)	0.060 (0.040–0.084)	1.947 (1.487–3.188)	0.521 (0.100–0.898)

LR, logistic regression; DT, decision tree; RF, random forest; XGBoost, eXtreme gradient boosting; SVM, support vector machine; AUC, area under receiver operating characteristic curve; F1, F1 score; Values are presented as point estimates with 95% confidence intervals.

**Figure 2:**
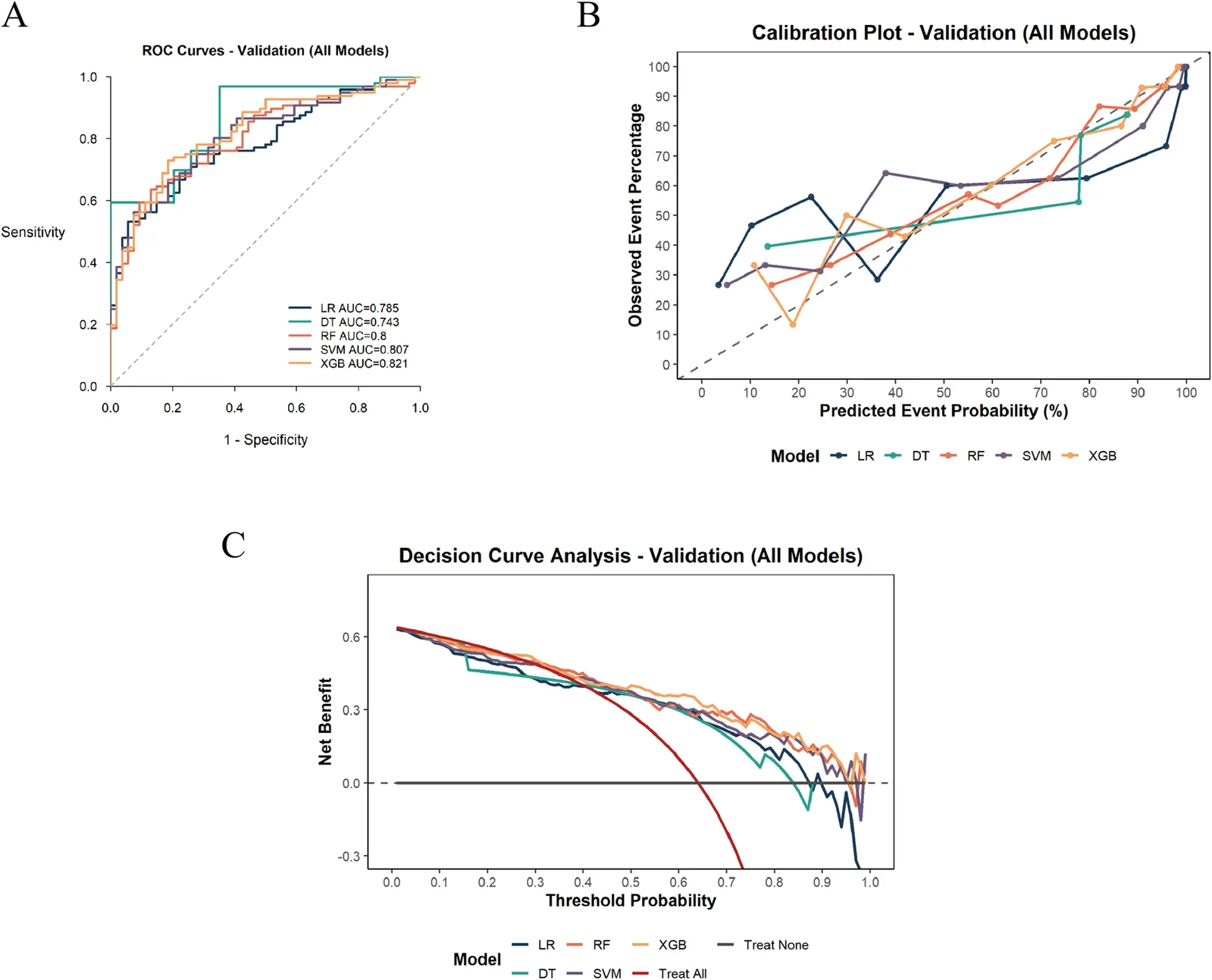
Performance of machine learning-based modeling framework in the internal validation set. **(A)** Receiver operating characteristic (ROC) curve showing the discriminative ability of the machine learning-based modeling framework. **(B)** Calibration curve comparing predicted probabilities to actual outcomes. **(C)** Decision curve analysis (DCA) curve illustrating the net clinical benefit of the machine learning-based modeling framework across different threshold probabilities.

### 3.4 External validation

The XGBoost model was subsequently applied to the external validation set. As shown in Table 3, the model achieved an AUC of 0.981 (95% CI: 0.963–0.994), with an accuracy of 0.920, sensitivity of 0.909, specificity of 0.935, precision of 0.952, and an F1-score of 0.930. The Brier score was 0.062 (95% bootstrap CI, 0.043–0.084), the calibration intercept was 0.150 (95% bootstrap CI: −0.210–0.473), and the calibration slope was 2.072 (95% bootstrap CI: 1.633–3.328). The calibration slope deviated substantially from the ideal value of 1, indicating that the dispersion of the predicted probabilities remained inconsistent with the observed outcome distribution. Therefore, adequate calibration should not be inferred solely from a low Brier score. The receiver operating characteristic curve, calibration plot, and decision curve for the external validation set are shown in Figures 3A–C.

**Figure 3:**
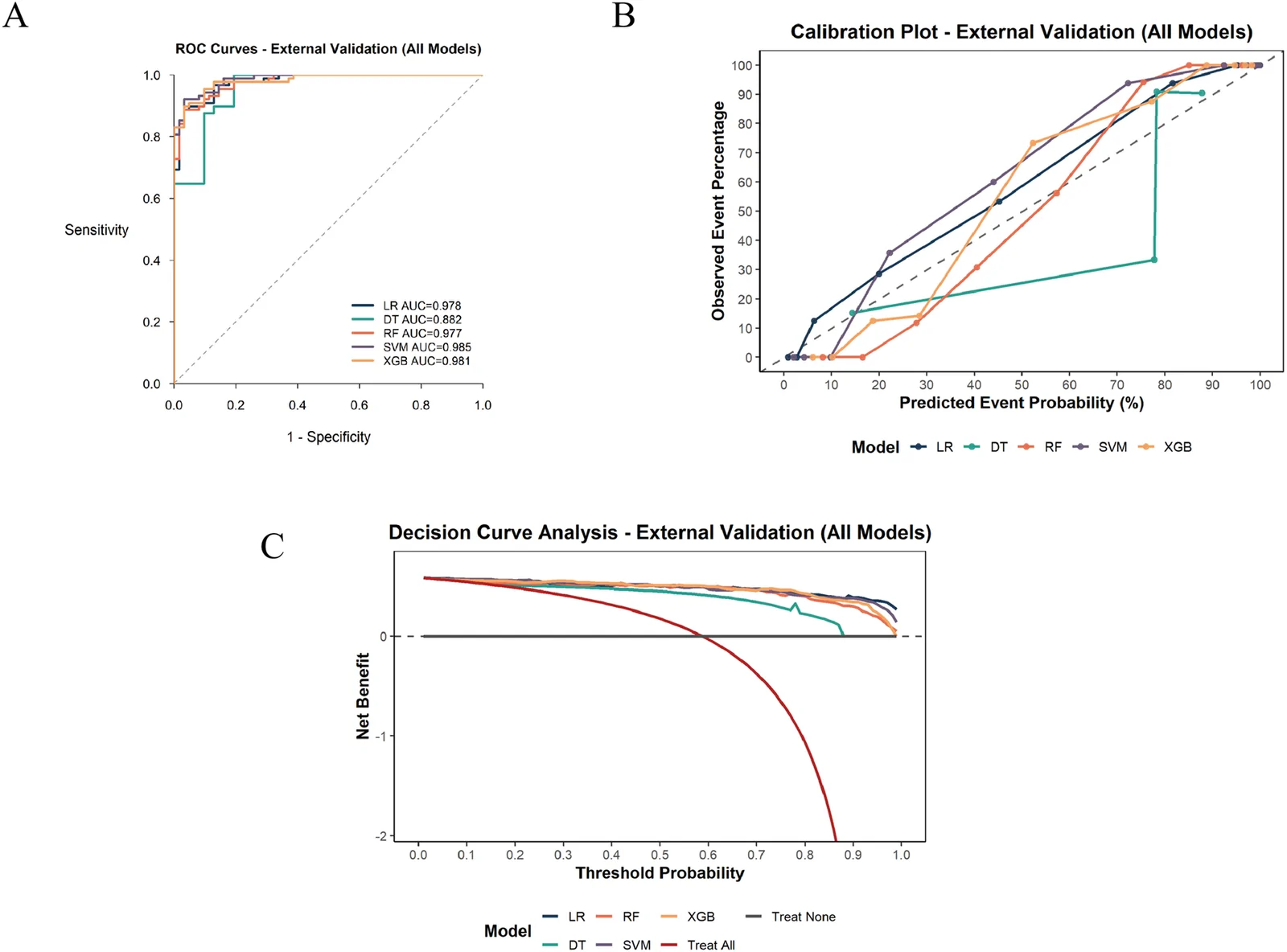
Performance of machine learning–based modeling framework in the external validation set. **(A)** ROC curve. **(B)** Calibration curve. **(C)** DCA curve.

Because the AUC in the external validation set was markedly higher than that in the internal validation set, additional analyses were performed to examine cohort shift and distributional differences across the datasets. The prevalence of kinesiophobia did not differ significantly among the development set, internal validation set, and external validation set (*P* = 0.503). However, differences in case mix between the external validation set and the development set were observed for several variables, including clinical classification, Barthel Index, resignation coping score, educational level, left ventricular end-diastolic volume (LVEDV), and left ventricular end-systolic volume (LVESV). In addition, outcome-specific distributions of predicted probabilities showed more pronounced separation in the external validation set than in the internal validation set.

In the external validation set, the median predicted probability was 0.144 [interquartile range (IQR): 0.079–0.255] among participants without kinesiophobia and 0.934 (IQR: 0.787–0.969) among those with kinesiophobia. The corresponding values in the internal validation set were 0.291 (IQR: 0.165–0.569) and 0.864 (IQR: 0.574–0.944), respectively. This more pronounced separation was consistent with the higher discrimination observed in the external validation set. Together with the observed differences in case mix, these findings suggest that the unusually high external AUC should be interpreted in the context of the distributional characteristics of the external cohort and should not be regarded as evidence that the model inherently performs better in external populations. Further validation in larger independent cohorts from different geographic regions and healthcare institutions is therefore warranted. Detailed results are presented in Supplementary Tables S8–S12.

### 3.5 SHAP analysis and interpretation

After algorithm selection and final model locking, SHapley Additive exPlanations (SHAP) analysis was performed to interpret the final XGBoost model. Based on the mean absolute SHAP values, the predictors were ranked in descending order of importance as follows: resignation coping (0.720), exercise self-efficacy (0.635), avoidance coping (0.380), fear of disease progression (0.342), depression (0.316), self-perceived burden (0.309), confrontation coping (0.156), gender (0.141), smoking status (0.135), residence (0.094), educational level (0.079), Barthel Index (0.039), clinical classification (0.022), drinking status (0.020), and pain severity (0.005). This ranking of predictor importance may help clinicians identify variables that contribute most strongly to the model's predictions and prioritize their assessment in clinical practice, thereby supporting more individualized clinical management. Detailed results are presented in Figure 4 and Supplementary Table 13.

**Figure 4:**
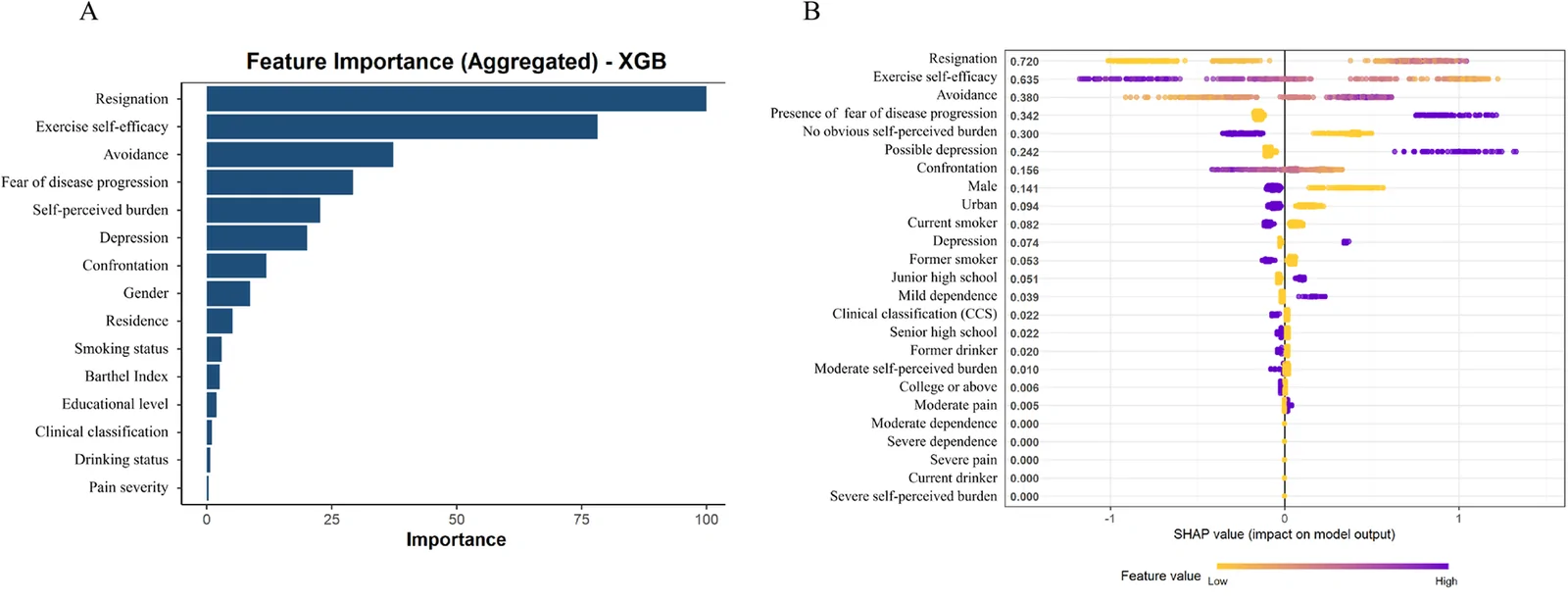
Feature importance analysis of the XGBoost model using the SHAP method. **(A)** SHAP summary bar plot showing feature importance based on mean absolute SHAP values. **(B)** SHAP summary dot plot showing the magnitude and direction of each feature's contribution to the model output.

## 4 Discussion

The initial internal cohort included 502 older patients with CHD who underwent PCI, of whom 321 had kinesiophobia, corresponding to a prevalence of 63.9%. This relatively high prevalence suggests that kinesiophobia represents an important barrier to participation in CR among older patients after PCI and warrants greater attention from healthcare professionals (23). Early identification of patients who may have kinesiophobia, together with the development of a reliable identification tool, may facilitate further clinical assessment and the formulation of individualized rehabilitation management strategies.

A rigorous nested cross-validation framework with 10-fold outer cross-validation and 5-fold inner cross-validation was used to compare five candidate algorithms: LR, DT, RF, SVM, and XGBoost. Within each resampling iteration, all data preprocessing, feature selection, SMOTE resampling, model fitting, and hyperparameter tuning were performed exclusively within the corresponding training partition to reduce the risk of information leakage associated with preprocessing, feature selection, and resampling. The outer loop was used to estimate model performance, whereas the inner loop was used for model selection and hyperparameter tuning. Under this nested cross-validation framework, the mean AUCs across the outer validation folds were 0.884 for LR, 0.795 for DT, 0.876 for RF, 0.879 for SVM, and 0.878 for XGBoost. The final algorithm was selected solely on the basis of a prespecified multimetric composite ranking derived from the inner cross-validation within the development set, without reference to the results of either the internal or external validation set. The composite evaluation simultaneously considered discrimination, overall probabilistic prediction error, calibration, and clinical net benefit. Although LR achieved a slightly higher mean AUC across the outer validation folds, XGBoost obtained the lowest composite rank sum during inner cross-validation and was therefore selected as the final algorithm. These findings underscore that algorithm selection should not rely on AUC alone but should incorporate multiple dimensions of model performance, including the Brier score, calibration intercept, calibration slope, and clinical net benefit. Neither the internal validation set nor the external validation set was used for algorithm selection or hyperparameter tuning, thereby avoiding *post hoc* model selection based on validation performance.

In the held-out internal validation set, the locked XGBoost model achieved an AUC of 0.821, indicating a certain degree of discriminative ability. The Brier score was 0.170, with a calibration intercept of 0.269, and a calibration slope of 0.744. The confidence interval for the calibration intercept included the ideal value of 0, and that for the calibration slope included the ideal value of 1; however, both intervals were relatively wide, indicating uncertainty in the calibration estimates, which may partly reflect the limited sample size of the internal validation set. Accordingly, the internal validation results support the predictive ability of the model, although they do not provide sufficient evidence to establish stable calibration performance across other clinical settings. The locked XGBoost model achieved an AUC of 0.981 in the external validation set, which was substantially higher than that observed in the internal validation set. However, the calibration slope in the external validation set was 2.072, with a 95% confidence interval of 1.633–3.328, markedly deviating from the ideal value of 1. Although the Brier score was low at 0.062, a low Brier score alone did not demonstrate adequate calibration. The Brier score was jointly influenced by both outcome prevalence and discriminative ability, whereas the calibration slope reflected the agreement between the dispersion of predicted probabilities and the observed outcome distribution. Therefore, the external validation results should be interpreted by integrating the AUC, Brier score, calibration intercept, calibration slope, and calibration plot, rather than judging model performance solely on the basis of a high AUC or a low Brier score. Further analyses of cohort shift showed no statistically significant difference in the prevalence of kinesiophobia among the development set, internal validation set, and external validation set. Thus, differences in outcome prevalence alone were unlikely to account for the substantially higher AUC observed in the external validation set. Nevertheless, certain differences in case mix were observed between the external validation set and the development set across clinical classification, Barthel Index, resignation coping score, educational level, LVEDV, and LVESV. The predicted probability distributions also showed greater separation between participants with and without kinesiophobia in the external validation set. Specifically, the median predicted probability was 0.144 among participants without kinesiophobia and 0.934 among those with kinesiophobia, compared with 0.291 and 0.864, respectively, in the internal validation set. This more pronounced separation provided a plausible statistical explanation for the higher AUC observed in the external validation set. However, the observed differences in case mix should not be interpreted as definitive evidence that they fully account for the elevated AUC in the external cohort. Several candidate algorithms also yielded high AUC values in the external validation set, suggesting that this phenomenon may be more closely related to characteristics of the external cohort, the strength of associations between predictors and the outcome, or the underlying data-generating process, rather than a unique merit of the XGBoost algorithm. In addition, the external validation set comprised 150 patients and was recruited from the same three hospitals as the development cohort, but with a distinct recruitment window. Accordingly, this cohort more accurately represents temporally separated validation rather than geographically independent external validation. Further independent validation in larger cohorts from diverse geographic regions and multiple healthcare institutions is therefore warranted.

Following rigorous nested cross-validation and algorithm selection, the feature-selection pipeline was refitted on the full development cohort, which identified 15 predictors for refitting the locked final model: Barthel Index, avoidance coping, residence, fear of disease progression, clinical classification, confrontation coping, resignation coping, pain severity, educational level, smoking status, gender, depression, drinking status, exercise self-efficacy, and self-perceived burden. Importantly, these 15 predictors represented the final feature set derived during model refitting on the full development cohort. SHAP analysis of the XGBoost model further revealed that the variables with the largest mean absolute SHAP values were dominated by resignation coping, exercise self-efficacy, avoidance coping, fear of disease progression, depression, and self-perceived burden. These results demonstrated that coping styles, exercise confidence, emotional status, and patients’ subjective disease-related burden perceptions exerted substantial predictive effects on model outputs. Nevertheless, SHAP values only quantified each predictor's marginal contribution to model predictions and did not reflect causal effects of these variables on kinesiophobia. Therefore, subsequent interpretations of individual predictors should be considered predictive associations rather than direct evidence of modifiable causal risk factors.

Resignation coping showed the greatest contribution among all predictors in the XGBoost model, and higher resignation coping scores were generally associated with a higher model-estimated probability of current kinesiophobia. This finding is consistent with previous studies in cardiovascular populations (24). A stronger tendency toward resignation coping may be associated with more negative perceptions of illness and exercise-related risk and may increase concerns that exercise could provoke cardiac discomfort or aggravate the underlying condition, thereby potentially contributing to reduced physical activity and lower participation in rehabilitation (25). The present study also showed that higher avoidance coping scores were associated with a higher model-estimated probability of current kinesiophobia, whereas higher confrontation coping scores were associated with a lower estimated probability. Persistent reliance on avoidance coping may reduce opportunities for patients to engage in exercise and rehabilitation, limiting their ability to modify maladaptive perceptions of exercise-related risk through direct experience and thereby potentially maintaining the interaction between fear and avoidance (26). In contrast, patients with a stronger tendency toward confrontation coping may be more inclined to actively seek disease- and rehabilitation-related information, address problems encountered during recovery, and develop more adaptive illness perceptions, appraisals of exercise-related risk, and rehabilitation expectations. Therefore, coping styles should be considered during cardiac rehabilitation assessment and education after PCI, with particular attention to patients showing pronounced resignation or avoidance coping tendencies, and with targeted psychological support and rehabilitation guidance provided when appropriate.

Exercise self-efficacy was also among the variables with a relatively high contribution in the XGBoost model. Previous studies have shown that exercise self-efficacy is closely associated with exercise motivation and short-term exercise behavior in patients with CHD, with higher levels of self-efficacy generally related to greater engagement in physical activity (27). Patients with lower exercise self-efficacy may have less confidence in their ability to perform rehabilitation exercise safely and may be more likely to develop concerns about exercise safety when experiencing palpitations, chest tightness, or other exercise-related discomfort. These concerns may be associated with a lower willingness to engage in physical activity. Accordingly, assessment of kinesiophobia and planning of CR after PCI should consider patients’ confidence in completing rehabilitation exercise, self-management capacity, and perceptions of exercise safety. Individualized health education and progressively graded exercise guidance may help strengthen patients’ confidence in exercising safely. Fear of disease progression was another variable with a relatively high contribution in the XGBoost model. It is characterized by persistent concerns regarding disease recurrence, deterioration, or progression and may be accompanied by heightened vigilance toward bodily symptoms and avoidance tendencies (28). In the present study, the prevalence of kinesiophobia was substantially higher among patients with dysfunctional fear of disease progression than among those without such dysfunction. This finding is consistent with recent evidence demonstrating close links between fear of disease progression and kinesiophobia in patients after PCI (29). A substantial proportion of participants in the present study were clinically classified as having acute coronary syndrome, and some may have experienced prominent symptoms such as chest pain. Such symptom experiences may increase concerns about disease recurrence and the safety of physical activity. Among patients with greater fear of disease progression, these concerns may extend to exercise-related situations, resulting in heightened vigilance toward exercise-induced discomfort or potential cardiovascular events and potentially reducing participation in physical activity or rehabilitation exercise. Therefore, CR assessment should consider concerns regarding disease progression, symptom changes, and exercise safety, with individualized health education and psychological support provided as appropriate. Self-perceived burden was also among the variables with a relatively high contribution in the model. Previous studies in patients with CHD after PCI have reported a positive association between self-perceived burden and kinesiophobia, consistent with the present findings (30). Patients with greater self-perceived burden may be more concerned about the caregiving, financial, and emotional strain that their illness and rehabilitation could place on their families and may consequently have greater concerns about participation in rehabilitation exercise. Such perceptions of burden may be associated with lower confidence in rehabilitation and reduced willingness to participate (31). Therefore, assessment of kinesiophobia may also include patients’ perceived burden and concerns about the impact of their illness on their families. Family support and psychosocial support may help identify and address psychological barriers that could interfere with participation in cardiac rehabilitation. Depression also showed a relatively high contribution in the XGBoost model. Previous studies have reported associations between kinesiophobia and anxiety and depressive symptoms in patients with myocardial infarction (32). Depressive symptoms may be accompanied by loss of interest, feelings of helplessness, and negative cognitive biases, which may increase sensitivity to palpitations, chest pain, or other bodily sensations during exercise and contribute to more negative interpretations of their potential consequences. These processes may be associated with greater exercise avoidance and lower levels of physical activity (33). Therefore, depressive symptoms and other emotional problems should also be considered when assessing kinesiophobia in older patients with coronary heart disease after PCI. Comprehensive assessment of emotional status and exercise-related concerns may help inform individualized psychological support and cardiac rehabilitation guidance. Residence, gender, and smoking status also contributed to predictions in the XGBoost model. Previous studies have reported higher levels of kinesiophobia among patients living in rural areas than among those living in urban areas, consistent with the present findings (34). This difference may be related to limited access to healthcare resources, insufficient coverage of cardiac rehabilitation education, and a lack of professional exercise guidance in rural areas, which may leave patients with greater uncertainty regarding the safety and appropriate intensity of exercise after PCI and greater concerns about exercise-related discomfort or complications. Female patients also exhibited higher levels of kinesiophobia, in line with previous studies (35). This pattern may be associated with greater sensitivity to exercise-related physical discomfort and more pronounced emotional distress, including anxiety and depressive symptoms, which may contribute to greater concerns about rehabilitation exercise (36). The predictive pattern observed for smoking status was not entirely consistent with some previous studies (37). Several explanations may account for this finding. First, a possible healthy-smoker or survivor-selection effect should be considered. Because the present study included older patients with established CHD who had undergone PCI, current smokers who remained eligible for participation may represent a selected subgroup with relatively preserved functional status or fewer perceived limitations (38, 39). Second, differences in health perceptions may contribute to this pattern. Some smokers may underestimate their personal vulnerability to health-related threats or perceive exercise-related symptoms differently, potentially resulting in lower self-reported fear of movement (40, 41). In addition, smoking status is closely related to demographic, behavioural, clinical, and psychosocial characteristics, and its predictive contribution may reflect complex correlations or interactions with factors such as sex, functional status, disease severity, and psychological status (38, 42, 43). Given the cross-sectional and predictive nature of this study, further research incorporating detailed smoking exposure and health-risk perception is needed to clarify the relationship between smoking status and kinesiophobia in older patients after PCI. Taken together, assessment of current kinesiophobia in older patients with CHD after PCI should extend beyond clinical characteristics to include coping styles, exercise self-efficacy, fear of disease progression, self-perceived burden, depression, and relevant demographic and behavioral characteristics. Incorporating psychological support, exercise-related health education, and progressively graded rehabilitation guidance into individualized CR programs may help identify and address modifiable psychological and behavioral factors associated with kinesiophobia and support greater participation in CR.

In clinical practice, the potential value of the final XGBoost model lies in assisting healthcare professionals in identifying older patients with coronary heart disease after PCI who may warrant further assessment for kinesiophobia, rather than replacing clinical judgment. The 15 variables included in the model encompass functional status, coping styles, emotional status, exercise self-efficacy, illness perceptions, and sociodemographic characteristics, providing a multidimensional basis for clinical assessment. For patients with lower exercise self-efficacy, greater reliance on avoidance or resignation coping, more severe depressive symptoms, or greater fear of disease progression, further standardized psychological assessment, education on exercise safety, and individualized stepwise rehabilitation guidance may be considered (44, 45). For patients living in areas with relatively limited healthcare resources, remote follow-up and telerehabilitation support may also be considered (46). However, the model should not be implemented as a routine clinical decision-support tool until its performance has been confirmed through broader independent external validation and, if necessary, recalibration.

Despite its strengths, this study has several limitations. First, all participants were recruited from three healthcare institutions within the same city. Although the external validation cohort was enrolled during a different time period, it was drawn from the same institutions, and its sample size was relatively limited. Therefore, the external validation results may have been influenced by regional characteristics, clinical practice patterns, and differences in case mix. Particularly, the unusually high external AUC and the calibration slope that deviated substantially from the ideal value indicated that the model's generalizability requires further evaluation in larger independent cohorts from different geographic regions and healthcare institutions. Second, although a rigorous nested cross-validation framework was used, with missing-data imputation, variable encoding, standardization, feature selection, SMOTE resampling, and hyperparameter optimization restricted to the corresponding training partitions to reduce information leakage and optimistic bias, the sample size of the development set remained relatively limited. The variables selected across different cross-validation folds were not entirely consistent, indicating some instability in the feature-selection process. Accordingly, the final set of 15 predictors identified using the full development set requires further validation in larger samples. Third, candidate predictors were primarily identified through literature review and expert discussion. Although the variables covered demographic, clinical, behavioral, and psychosocial domains, other potentially important factors associated with kinesiophobia may not have been captured. In addition, several predictors were derived from patient-reported measures and may therefore have been affected by recall bias, social desirability bias, or measurement error. Future studies should consider incorporating more objective measures, such as physical activity monitoring, exercise capacity, socioeconomic characteristics, and participation in cardiac rehabilitation. Fourth, this study used a cross-sectional design, with predictors and kinesiophobia status assessed at the same time point. Accordingly, the model estimates the probability of concurrent kinesiophobia at the time of assessment and cannot predict the long-term occurrence of kinesiophobia or establish the temporal sequence or causal relationships between the predictors and kinesiophobia. Kinesiophobia may change over the course of rehabilitation as symptoms improve and psychological or behavioral interventions are introduced. Longitudinal follow-up and temporal validation are therefore needed to evaluate the stability of model performance over time and to explore the development of dynamic prediction approaches. Overall, this study redeveloped the prediction model using a rigorous nested cross-validation framework and selected XGBoost as the final algorithm on the basis of a prespecified multimetric composite evaluation conducted within the development set. The final model demonstrated moderate discrimination in the internal validation cohort and a high AUC in the external validation cohort. However, the calibration slope observed in the external cohort, together with evidence of cohort shift, indicates that the external performance should be interpreted cautiously. The model shows potential as an adjunctive tool for identifying older patients after PCI who may warrant further assessment for kinesiophobia; however, its calibration, stability, and clinical transportability require further confirmation in larger, geographically independent, prospective cohorts.

## 5 Conclusion

This study developed an interpretable machine learning model for the concurrent identification of kinesiophobia in older patients with CHD after PCI and temporally validated it in an external cohort. Following a prespecified feature-selection procedure, 15 predictors were retained for final model refitting. Among the candidate algorithms, XGBoost was selected as the final model on the basis of a prespecified multimetric composite evaluation incorporating discrimination, overall probabilistic prediction error, calibration, and clinical net benefit. SHAP analysis further characterized the contributions of individual predictors to model predictions, thereby enhancing model interpretability. The final model demonstrated moderate discrimination in the internal validation cohort and high discrimination in the temporally distinct external validation cohort. However, the external calibration slope and observed differences between cohorts indicate that the external validation results should be interpreted cautiously. The model may serve as an adjunctive tool to help healthcare professionals identify patients who warrant further assessment for kinesiophobia. Broader independent external validation and, where necessary, recalibration are required before routine clinical implementation.

## Data Availability

The raw data supporting the conclusions of this article will be made available by the authors, without undue reservation.
