# Supplementary material for: Development and validation of an interpretable machine learning model for concurrent identification of kinesiophobia in older patients with coronary heart disease after percutaneous coronary intervention
Source: Front Med (Lausanne). 2026 Sep 23;13:1915056. doi: 10.3389/fmed.2026.1915056 (PMC13642195; doi:10.3389/fmed.2026.1915056)
Supplement: Supplementary file 1 [file Datasheet1.docx]

**Development and validation of an interpretable machine learning model for concurrent identification of kinesiophobia in older patients with coronary heart disease after percutaneous coronary intervention**

Yaya Xi ^a,1^, Meishan Zhang ^a,1^, Leyao Han ^a,2^, Minjie Liu ^a,3^, Jingying Xiong ^a,4^, Yunyun Liu ^a,5^, Xinman Dou ^d,a,6^, Bo Wan ^b,7^, Haixia Wang ^c,8^, Xinglei Wang ^b,a^*

^a^ School of Nursing, Lanzhou University, Lanzhou, China.

^b^ Department of Cardiovascular Medicine, Lanzhou University Second Hospital, Lanzhou, China.

^c^ The Sixth Ward of General Surgery, Lanzhou University Second Hospital, Lanzhou, Gansu Province, China.

^d^ Department of Nursing, Lanzhou University Second Hospital, Lanzhou, China.

*** Correspondence:**
Xinglei Wang, Department of Cardiovascular Medicine, Lanzhou University Second Hospital; School of Nursing, Lanzhou University, Lanzhou, China.
1106087304@qq.com

**^1^ Co-first Authorship:** Yaya Xi and Meishan Zhang contributed equally to this manuscript.

**Supplementary Figure 1.** Study workflow.

Supplementary Table 1. The proportion of missing values for variables in the development set

| Variables | Missing Values (%) |
| --- | --- |
| LVEDV | 3.41 |
| LVESV | 3.41 |
| LAD | 3.41 |
| LVEF | 2.84 |
| TC | 0.28 |

TC: total cholesterol; LVEF: left ventricular ejection fraction; LVEDV: left ventricular end-diastolic volume; LVESV: left ventricular end-systolic volume; LAD: left atrial diameter; Candidate variables not listed in this table had a missing data rate of 0%, and were therefore omitted.

Supplementary Table 2. The proportion of missing values for variables in

internal validation set.

| Variables | Missing Values (%) |
| --- | --- |
| TC | 0.67 |
| LVEDV | 0.67 |
| LVESV | 0.67 |
| LAD | 0.67 |

TC: total cholesterol; LVEDV: left ventricular end-diastolic volume; LVESV: left ventricular end-systolic volume; LAD: left atrial diameter; Candidate variables not listed in this table had a missing data rate of 0%, and were therefore omitted.

Supplementary Table 3. The proportion of missing values for variables in

external validation set.

| Variables | Missing Values (%) |
| --- | --- |
| LVEF | 3.33 |
| LVEDV | 3.33 |
| LVESV | 3.33 |
| LAD | 3.33 |
| TC | 1.33 |

TC: total cholesterol; LVEF: left ventricular ejection fraction; LVEDV: left ventricular end-diastolic volume; LVESV: left ventricular end-systolic volume; LAD: left atrial diameter; Candidate variables not listed in this table had a missing data rate of 0%, and were therefore omitted.

Supplementary Table 4. The missing data imputation methods used during final refitting on the full development set.

| Variable | Imputation method |
| --- | --- |
| TC | median |
| LVEF | mode |
| LVEDV | median |
| LVESV | median |
| LAD | median |

TC: total cholesterol; LVEF: left ventricular ejection fraction; LVEDV: left ventricular end-diastolic volume; LVESV: left ventricular end-systolic volume; LAD: left atrial diameter.

Supplementary Table 5. Characteristics of the External Validation Set.

| Variables | Kinesiophobia group (n = 88) | Non-kinesiophobia group (n = 62) |
| --- | --- | --- |
| Age, Median (IQR) | 65.5 (61.0, 70.0) | 63.0 (60.0, 68.3) |
| BMI, (Mean ± SD) | 23.70 ± 3.362 | 24.43 ± 2.625 |
| Gender, n (%) |  |  |
| Male | 65 (54.2) | 55 (45.8) |
| Female | 23 (76.7) | 7 (23.3) |
| Marital status, n (%) |  |  |
| Married | 83 (59.3) | 57 (40.7) |
| Unmarried | 0 (0.0) | 0 (0.0) |
| Widowed | 4 (44.4) | 5 (55.6) |
| Divorced | 1 (100.0) | 0 (0.0) |
| Educational level, n (%) |  |  |
| Primary school or below | 30 (66.7) | 15 (33.3) |
| Junior high school | 25 (78.1) | 7 (21.9) |
| Senior high school/technical secondary school | 22 (44.9) | 27 (55.1) |
| College or above | 11 (45.8) | 13 (54.2) |
| Residence, n (%) |  |  |
| Rural | 34 (61.8) | 21 (38.2) |
| Urban | 54 (56.8) | 41 (43.2) |
| Physical activity level, n (%) |  |  |
| Low | 61 (81.3) | 14 (18.7) |
| Moderate | 23 (37.1) | 39 (62.9) |
| High | 4 (30.8) | 9 (69.2) |
| Sedentary behaviour, n (%) |  |  |
| No | 27 (40.9) | 39 (59.1) |
| Yes | 61 (72.6) | 23 (27.4) |
| Drinking status, n (%) |  |  |
| Never drinker | 46 (59.7) | 31 (40.3) |
| Former drinker | 18 (60.0) | 12 (40.0) |
| Current drinker | 24 (55.8) | 19 (44.2) |
| Smoking status, n (%) |  |  |
| Never smoker | 36 (64.3) | 20 (35.7) |
| Former smoker | 21 (60.0) | 14 (40.0) |
| Current smoker | 31 (52.5) | 28 (47.5) |
| Time since PCI (days), Median (IQR) | 1 (1, 1) | 1 (1, 1) |
| TC (mmol/L), Median (IQR) | 3.67 (2.98, 4.23) | 3.34 (2.85, 3.89) |
| LVEDV (ml), Median (IQR) | 112.0 (90.3, 122.0) | 107.5 (91.3, 122.0) |
| LVESV (ml), Median (IQR) | 42.5 (33.0, 57.0) | 38.5 (33.8, 50.0) |
| LAD (mm), Median (IQR) | 35.0 (32.0, 38.0) | 34.0 (32.0, 37.0) |
| Medical Coping Modes, Median (IQR) |  |  |
| Confrontation | 15.0 (13.0, 17.0) | 20.0 (16.0, 23.0) |
| Avoidance | 18.0 (16.0, 19.0) | 12.0 (11.0, 14.0) |
| Resignation | 7.5 (6.0, 10.0) | 5.0 (5.0, 6.0) |
| Exercise self-efficacy, Median (IQR) | 2.33 (1.22, 4.31) | 9.00 (7.78, 9.56) |
| Number of PCI procedures, n (%) |  |  |
| 1 | 53 (66.3) | 27 (33.8) |
| 2 | 27 (48.2) | 29 (51.8) |
| ≥3 | 8 (57.1) | 6 (42.9) |
| Procedure type, n (%) |  |  |
| Elective | 84 (57.9) | 61 (42.1) |
| Emergency | 4 (80.0) | 1 (20.0) |
| Puncture site, n (%) |  |  |
| Radial artery | 84 (57.9) | 61 (42.1) |
| Femoral artery | 4 (80.0) | 1 (20.0) |
| Number of comorbidities, n (%) |  |  |
| 0 | 23 (63.9) | 13 (36.1) |
| 1 | 33 (52.4) | 30 (47.6) |
| 2 | 25 (73.5) | 9 (26.5) |
| ≥3 | 7 (41.2) | 10 (58.8) |
| Pain severity, n (%) |  |  |
| Mild pain | 74 (54.8) | 61 (45.2) |
| Moderate pain | 14 (93.3) | 1 (6.7) |
| Severe pain | 0 (0.0) | 0 (0.0) |
| Clinical classification, n (%) |  |  |
| CCS | 32 (57.1) | 24 (42.9) |
| ACS | 56 (59.6) | 38 (40.4) |
| LVEF (%), n (%) |  |  |
| ＜40 | 4 (100.0) | 0 (0.0) |
| 40～49 | 9 (90.0) | 1 (10.0) |
| ≥50 | 75 (55.1) | 61 (44.9) |
| Anxiety, n (%) |  |  |
| No anxiety | 54 (47.8) | 59 (52.2) |
| Possible anxiety | 20 (90.9) | 2 (9.1) |
| Anxiety | 14 (93.3) | 1 (6.7) |
| Depression, n (%) |  |  |
| No depression | 30 (33.0) | 61 (67.0) |
| Possible depression | 27 (96.4) | 1 (3.6) |
| Depression | 31 (100.0) | 0 (0.0) |
| Self-perceived burden, n (%) |  |  |
| No obvious burden | 32 (39.5) | 49 (60.5) |
| Mild burden | 40 (78.4) | 11 (21.6) |
| Moderate burden | 16 (88.9) | 2 (11.1) |
| Severe burden | 0 (0.0) | 0 (0.0) |
| Barthel Index, n (%) |  |  |
| Independent | 49 (45.8) | 58 (54.2) |
| Mild dependence | 37 (90.2) | 4 (9.8) |
| Moderate dependence | 1 (100.0) | 0 (0.0) |
| Severe dependence | 1 (100.0) | 0 (0.0) |
| Fear of disease progression, n (%) |  |  |
| Present | 32 (86.5) | 5 (13.5) |
| Absent | 56 (49.6) | 57 (50.4) |

BMI: body mass index; TC: total cholesterol; LVEF: left ventricular ejection fraction; LVEDV: left ventricular end-diastolic volume; LVESV: left ventricular end-systolic volume; LAD: left atrial diameter; ACS: acute coronary syndrome; CCS: chronic coronary syndrome; IQR: interquartile range; For categorical variables, percentages in the kinesiophobia and non-kinesiophobia groups are row percentages, calculated using the total number of patients within each category level as the denominator. Percentages may not sum exactly to 100.0% due to rounding.

Supplementary Table 6. Comparison of baseline characteristics among the development, internal validation, and external validation cohorts.

| Variables | Development set  (n = 352) | Internal validation set (n = 150) | External validation set (n = 150) | Statistical value | *P* |
| --- | --- | --- | --- | --- | --- |
| Age, Median (IQR) | 63.00 (60.00, 70.25) | 63.00 (60.00, 70.00) | 64.00 (61.00, 70.00) | 1.537^a^ | 0.464 |
| BMI, (Mean ± SD) | 24.43 ± 3.13 | 24.47 ± 3.07 | 24.00 ± 3.09 | 1.159^c^ | 0.314 |
| Gender, n (%) |  |  |  | 1.386^b^ | 0.500 |
| Male | 267 (75.9) | 112 (74.7) | 120 (80.0) |  |  |
| Female | 85 (24.1) | 38 (25.3) | 30 (20.0) |  |  |
| Marital status, n (%) |  |  |  | — | 0.977^d^ |
| Married | 328 (93.2) | 140 (93.3) | 140 (93.3) |  |  |
| Unmarried | 2 (0.6) | 1 (0.7) | 0 (0.0) |  |  |
| Widowed | 21 (6.0) | 8 (5.3) | 9 (6.0) |  |  |
| Divorced | 1 (0.3) | 1 (0.7) | 1 (0.7) |  |  |
| Educational level, n (%) |  |  |  | 9.214^b^ | 0.162 |
| Primary school or below | 111 (31.5) | 56 (37.3) | 45 (30.0) |  |  |
| Junior high school | 107 (30.4) | 43 (28.7) | 32 (21.3) |  |  |
| Senior high school/technical secondary school | 90 (25.6) | 33 (22.0) | 49 (32.7) |  |  |
| College or above | 44 (12.5) | 18 (12.0) | 24 (16.0) |  |  |
| Residence, n (%) |  |  |  | 0.840^b^ | 0.657 |
| Rural | 144 (40.9) | 61 (40.7) | 55 (36.7) |  |  |
| Urban | 208 (59.1) | 89 (59.3) | 95 (63.3) |  |  |
| Physical activity level, n (%) |  |  |  | 2.567^b^ | 0.633 |
| Low | 193 (54.8) | 76 (50.7) | 75 (50.0) |  |  |
| Moderate | 134 (38.1) | 58 (38.7) | 62 (41.3) |  |  |
| High | 25 (7.1) | 16 (10.7) | 13 (8.7) |  |  |
| Sedentary behaviour, n (%) |  |  |  | 0.860^b^ | 0.651 |
| No | 165 (46.9) | 74 (49.3) | 66 (44.0) |  |  |
| Yes | 187 (53.1) | 76 (50.7) | 84 (56.0) |  |  |
| Drinking status, n (%) |  |  |  | 3.885^b^ | 0.422 |
| Never drinker | 175 (49.7) | 80 (53.3) | 77 (51.3) |  |  |
| Former drinker | 94 (26.7) | 32 (21.3) | 30 (20.0) |  |  |
| Current drinker | 83 (23.6) | 38 (25.3) | 43 (28.7) |  |  |
| Smoking status, n (%) |  |  |  | 1.283^b^ | 0.864 |
| Never smoker | 134 (38.1) | 63 (42.0) | 56 (37.3) |  |  |
| Former smoker | 82 (23.3) | 36 (24.0) | 35 (23.3) |  |  |
| Current smoker | 136 (38.6) | 51 (34.0) | 59 (39.3) |  |  |
| Time since PCI (days), Median (IQR) | 1.00 (1.00, 1.00) | 1.00 (1.00, 1.00) | 1.00 (1.00, 1.00) | 0.051^a^ | 0.975 |
| TC (mmol/L), Median (IQR) | 3.53 (2.99, 4.38) | 3.59 (2.95, 4.60) | 3.46 (2.94, 4.16) | 0.988^a^ | 0.610 |
| LVEDV (ml), Median (IQR) | 100.00 (86.00, 117.00) | 93.50 (82.00, 113.75) | 110.00 (91.00, 122.00) | 16.981^a^ | < 0.001 |
| LVESV (ml), Median (IQR) | 37.00 (29.00, 47.00) | 35.00 (28.00, 49.75) | 41.00 (33.25, 52.00) | 11.765^a^ | 0.003 |
| LAD (mm), Median (IQR) | 34.00 (31.75, 37.00) | 34.00 (31.00, 38.00) | 35.00 (32.00, 37.75) | 0.784^a^ | 0.676 |
| Medical Coping Modes, Median (IQR) |  |  |  |  |  |
| Confrontation | 17.00 (13.75, 19.00) | 17.00 (13.00, 19.00) | 16.00 (13.00, 20.00) | 0.078^a^ | 0.962 |
| Avoidance | 16.00 (13.00, 18.00) | 16.00 (14.00, 18.00) | 16.00 (12.00, 18.00) | 2.425^a^ | 0.297 |
| Resignation | 7.00 (5.00, 10.00) | 7.00 (5.00, 10.00) | 6.00 (5.00, 9.00) | 7.101^a^ | 0.029 |
| Exercise self-efficacy, Median (IQR) | 5.22 (3.11, 7.89) | 5.17 (3.03, 8.00) | 4.61 (1.92, 8.75) | 0.675^a^ | 0.714 |
| Number of PCI procedures, n (%) |  |  |  | 3.172^b^ | 0.529 |
| 1 | 211 (59.9) | 90 (60.0) | 80 (53.3) |  |  |
| 2 | 115 (32.7) | 45 (30.0) | 56 (37.3) |  |  |
| ≥3 | 26 (7.4) | 15 (10.0) | 14 (9.3) |  |  |
| Procedure type, n (%) |  |  |  | 3.729^b^ | 0.155 |
| Elective | 327 (92.9) | 144 (96.0) | 145 (96.7) |  |  |
| Emergency | 25 (7.1) | 6 (4.0) | 5 (3.3) |  |  |
| Puncture site, n (%) |  |  |  | 4.639^b^ | 0.098 |
| Radial artery | 322 (91.5) | 137 (91.3) | 145 (96.7) |  |  |
| Femoral artery | 30 (8.5) | 13 (8.7) | 5 (3.3) |  |  |
| Number of comorbidities, n (%) |  |  |  | 2.017^b^ | 0.918 |
| 0 | 87 (24.7) | 43 (28.7) | 36 (24.0) |  |  |
| 1 | 148 (42.0) | 59 (39.3) | 63 (42.0) |  |  |
| 2 | 85 (24.1) | 32 (21.3) | 34 (22.7) |  |  |
| ≥3 | 32 (9.1) | 16 (10.7) | 17 (11.3) |  |  |
| Pain severity, n (%) |  |  |  | — | 0.178^d^ |
| Mild pain | 310 (88.1) | 126 (84.0) | 135 (90.0) |  |  |
| Moderate pain | 37 (10.5) | 19 (12.7) | 15 (10.0) |  |  |
| Severe pain | 5 (1.4) | 5 (3.3) | 0 (0.0) |  |  |
| Clinical classification, n (%) |  |  |  | 37.284^b^ | < 0.001 |
| ACS | 291 (82.7) | 134 (89.3) | 94 (62.7) |  |  |
| CCS | 61 (17.3) | 16 (10.7) | 56 (37.3) |  |  |
| LVEF (%), n (%) |  |  |  | 3.072^b^ | 0.546 |
| <40 | 16 (4.5) | 4 (2.7) | 4 (2.7) |  |  |
| 40～49 | 22 (6.2) | 14 (9.3) | 10 (6.7) |  |  |
| ≥50 | 314 (89.2) | 132 (88.0) | 136 (90.7) |  |  |
| Anxiety, n (%) |  |  |  | 2.183^b^ | 0.702 |
| No anxiety | 263 (74.7) | 104 (69.3) | 113 (75.3) |  |  |
| Possible anxiety | 48 (13.6) | 26 (17.3) | 22 (14.7) |  |  |
| Anxiety | 41 (11.6) | 20 (13.3) | 15 (10.0) |  |  |
| Depression, n (%) |  |  |  | 4.568^b^ | 0.335 |
| No depression | 239 (67.9) | 92 (61.3) | 91 (60.7) |  |  |
| Possible depression | 60 (17.0) | 33 (22.0) | 28 (18.7) |  |  |
| Depression | 53 (15.1) | 25 (16.7) | 31 (20.7) |  |  |
| Self-perceived burden, n (%) |  |  |  | — | 0.666^e^ |
| No obvious burden | 175 (49.7) | 76 (50.7) | 81 (54.0) |  |  |
| Mild burden | 125 (35.5) | 48 (32.0) | 51 (34.0) |  |  |
| Moderate burden | 46 (13.1) | 24 (16.0) | 18 (12.0) |  |  |
| Severe burden | 6 (1.7) | 2 (1.3) | 0 (0.0) |  |  |
| Barthel Index, n (%) |  |  |  | — | 0.005^d^ |
| Independent | 288 (81.8) | 117 (78.0) | 107 (71.3) |  |  |
| Mild dependence | 50 (14.2) | 24 (16.0) | 41 (27.3) |  |  |
| Moderate dependence | 12 (3.4) | 6 (4.0) | 1 (0.7) |  |  |
| Severe dependence | 2 (0.6) | 3 (2.0) | 1 (0.7) |  |  |
| Fear of disease progression, n (%) |  |  |  | 0.096^b^ | 0.953 |
| Present | 89 (25.3) | 36 (24.0) | 37 (24.7) |  |  |
| Absent | 263 (74.7) | 114 (76.0) | 113 (75.3) |  |  |

BMI: body mass index; TC: total cholesterol; LVEF: left ventricular ejection fraction; LVEDV: left ventricular end-diastolic volume; LVESV: left ventricular end-systolic volume; LAD: left atrial diameter; ACS: acute coronary syndrome; CCS: chronic coronary syndrome; IQR: interquartile range; -, no value available; ᵃ H statistic for the Kruskal–Wallis test; ᵇ χ² statistic for Pearson’s chi-square test; ^c^ F statistic for one-way ANOVA ; ^d^ P value from Fisher’s exact test; ^e^ *P* value from Fisher’s exact test with Monte Carlo simulation using 10,000 replicates.

Supplementary Table 7. Multi-metric ranking and algorithm selection based on inner cross-validation in the development set

| Model | AUC | Brier | Calibration Intercept | Calibration Slope | Mean Net Benefit | Composite Rank Sum |
| --- | --- | --- | --- | --- | --- | --- |
| XGBoost | 0.877 (2) | 0.140 (1) | 0.488 (2) | 0.971 (1) | 0.512 (1) | 7 |
| RF | 0.864 (4) | 0.145 (2) | 0.317 (1) | 0.886 (2) | 0.511 (2) | 11 |
| SVM | 0.878 (1) | 0.145 (3) | 0.532 (3) | 0.749 (3) | 0.504 (3) | 13 |
| LR | 0.873 (3) | 0.150 (4) | 0.636 (4) | 0.566 (5) | 0.500 (4) | 20 |
| DT | 0.813 (5) | 0.175 (5) | 0.674 (5) | 0.726 (4) | 0.479 (5) | 24 |

LR: Logistic Regression; DT: Decision Tree; RF: Random Forest; XGBoost: eXtreme Gradient Boosting; SVM: Support Vector Machine; AUC: area under receiver operating characteristic curve; A lower composite rank sum indicates better overall performance.

Supplementary Table 8. Univariable associations between selected predictors and kinesiophobia in the development, internal validation, and external validation sets

| **Variable** | Development OR  (95% CI) | *P* | Internal validation OR (95% CI) | *P* | External validation OR (95% CI) | *P* |
| --- | --- | --- | --- | --- | --- | --- |
| **Gender** |  |  |  |  |  |  |
| **Male** | 0.297 (0.156–0.532) | < 0.001 | 0.654 (0.285–1.423) | 0.296 | 0.360 (0.134–0.864) | 0.029 |
| **Educational level** |  |  |  |  |  |  |
| **Junior high school** | 0.908 (0.510–1.617) | 0.743 | 0.799 (0.346–1.850) | 0.599 | 1.786 (0.645–5.313) | 0.276 |
| **Senior high school/technical secondary school** | 0.579 (0.322–1.036) | 0.066 | 0.947 (0.381–2.409) | 0.908 | 0.407 (0.173–0.931) | 0.036 |
| **College or above** | 0.423 (0.205–0.866) | 0.019 | 0.474 (0.158–1.405) | 0.175 | 0.423 (0.150–1.159) | 0.096 |
| **Residence** |  |  |  |  |  |  |
| **Urban** | 0.565 (0.356–0.886) | 0.014 | 0.891 (0.447–1.755) | 0.740 | 0.813 (0.409–1.597) | 0.551 |
| **Medical Coping Modes** |  |  |  |  |  |  |
| **Confrontation** | 0.872 (0.822–0.924) | < 0.001 | 0.918 (0.840–1.000) | 0.054 | 0.772 (0.698–0.845) | < 0.001 |
| **Avoidance** | 1.361 (1.253–1.489) | < 0.001 | 1.170 (1.038–1.330) | 0.013 | 2.421 (1.911–3.279) | < 0.001 |
| **Resignation** | 1.554 (1.389–1.760) | < 0.001 | 1.327 (1.141–1.571) | < 0.001 | 1.762 (1.420–2.277) | < 0.001 |
| **Exercise self-efficacy** | 0.652 (0.585–0.721) | < 0.001 | 0.678 (0.579–0.784) | < 0.001 | 0.449 (0.348–0.550) | < 0.001 |
| **Pain severity** |  |  |  |  |  |  |
| **Moderate pain** | 4.153 (1.713–12.407) | < 0.001 | 1.782 (0.637–5.794) | 0.295 | 11.541 (2.224–212.080) | 0.020 |
| **Clinical classification** |  |  |  |  |  |  |
| **CCS** | 0.517 (0.295–0.904) | 0.020 | 0.695 (0.243–2.055) | 0.496 | 0.905 (0.463–1.777) | 0.770 |
| **Smoking status** |  |  |  |  |  |  |
| **Former smoker** | 0.334 (0.183–0.603) | < 0.001 | 0.967 (0.418–2.268) | 0.938 | 0.833 (0.349–2.000) | 0.681 |
| **Current smoker** | 0.376 (0.220–0.635) | < 0.001 | 1.346 (0.620–2.971) | 0.455 | 0.615 (0.288–1.294) | 0.203 |
| **Drinking status** |  |  |  |  |  |  |
| **Former drinker** | 0.455 (0.268–0.769) | < 0.001 | 1.086 (0.465–2.628) | 0.852 | 1.011 (0.430–2.432) | 0.980 |
| **Current drinker** | 0.435 (0.251–0.750) | < 0.001 | 0.975 (0.440–2.204) | 0.950 | 0.851 (0.400–1.820) | 0.676 |
| **Depression** |  |  |  |  |  |  |
| **Possible depression** | 31.270 (9.462–193.488) | < 0.001 | 5.600 (2.140–17.625) | < 0.001 | 54.900 (10.877–1002.757) | < 0.001 |
| **Self-perceived burden** |  |  |  |  |  |  |
| **No obvious burden** | 0.272 (0.162–0.448) | < 0.001 | 0.241 (0.103–0.528) | < 0.001 | 0.180 (0.078–0.390) | < 0.001 |
| **Moderate burden** | 4.526 (1.505–19.594) | 0.017 | 6.838 (1.208–129.135) | 0.074 | 2.200 (0.514–15.251) | 0.338 |
| **Barthel Index** |  |  |  |  |  |  |
| **Mild dependence** | 4.143 (1.912–10.361) | < 0.001 | 3.601 (1.268–12.974) | 0.029 | 10.949 (4.042–38.469) | < 0.001 |
| **Fear of disease progression** | 14.536 (6.282–42.314) | < 0.001 | 14.258 (4.070–90.474) | < 0.001 | 6.514 (2.557–20.126) | < 0.001 |

CCS: chronic coronary syndrome; OR: odds ratio; CI: confidence interval.

Supplementary Table 9. Distribution of the final predictors across the development, internal validation, and external validation sets

| Variable | Development set (n = 352) | Internal validation set (n = 150) | External validation set (n = 150) |
| --- | --- | --- | --- |
| **Gender**, n (%) |  |  |  |
| Male | 267 (75.9) | 112 (74.7) | 120 (80.0) |
| Female | 85 (24.1) | 38 (25.3) | 30 (20.0) |
| **Educational level**, n (%) |  |  |  |
| Primary school or below | 111 (31.5) | 56 (37.3) | 45 (30.0) |
| Junior high school | 107 (30.4) | 43 (28.7) | 32 (21.3) |
| Senior high school/technical secondary school | 90 (25.6) | 33 (22.0) | 49 (32.7) |
| College or above | 44 (12.5) | 18 (12.0) | 24 (16.0) |
| **Residence**, n (%) |  |  |  |
| Urban | 208 (59.1) | 89 (59.3) | 95 (63.3) |
| Rural | 144 (40.9) | 61 (40.7) | 55 (36.7) |
| **Medical Coping Modes**, Median (IQR) |  |  |  |
| Confrontation | 17.00 (13.75,19.00) | 17.00 (13.00,19.00) | 16.00 (13.00,20.00) |
| Avoidance | 16.00 (13.00,18.00) | 16.00 (14.00,18.00) | 16.00 (12.00,18.00) |
| Resignation | 7.00 (5.00,10.00) | 7.00 (5.00,10.00) | 6.00 (5.00,9.00) |
| **Exercise self-efficacy**, Median (IQR) | 5.22 (3.11,7.89) | 5.17 (3.03,8.00) | 4.61 (1.92,8.75) |
| **Pain severity**, n (%) |  |  |  |
| Mild pain | 310 (88.1) | 126 (84.0) | 135 (90.0) |
| Moderate pain | 37 (10.5) | 19 (12.7) | 15 (10.0) |
| Severe pain | 5 (1.4) | 5 (3.3) | 0 (0.0) |
| **Clinical classification**, n (%) |  |  |  |
| ACS | 291 (82.7) | 134 (89.3) | 94 (62.7) |
| CCS | 61 (17.3) | 16 (10.7) | 56 (37.3) |
| **Smoking status**, n (%) |  |  |  |
| Never smoker | 134 (38.1) | 63 (42.0) | 56 (37.3) |
| Former smoker | 82 (23.3) | 36 (24.0) | 35 (23.3) |
| Current smoker | 136 (38.6) | 51 (34.0) | 59 (39.3) |
| **Drinking status**, n (%) |  |  |  |
| Never drinker | 175 (49.7) | 80 (53.3) | 77 (51.3) |
| Former drinker | 94 (26.7) | 32 (21.3) | 30 (20.0) |
| Current drinker | 83 (23.6) | 38 (25.3) | 43 (28.7) |
| **Depression**, n (%) |  |  |  |
| No depression | 239 (67.9) | 92 (61.3) | 91 (60.7) |
| Possible depression | 60 (17.0) | 33 (22.0) | 28 (18.7) |
| Depression | 53 (15.1) | 25 (16.7) | 31 (20.7) |
| **Self-perceived burden**, n (%) |  |  |  |
| No obvious burden | 175 (49.7) | 76 (50.7) | 81 (54.0) |
| Mild burden | 125 (35.5) | 48 (32.0) | 51 (34.0) |
| Moderate burden | 46 (13.1) | 24 (16.0) | 18 (12.0) |
| Severe burden | 6 (1.7) | 2 (1.3) | 0 (0.0) |
| **Barthel Index**, n (%) |  |  |  |
| Independent | 288 (81.8) | 117 (78.0) | 107 (71.3) |
| Mild dependence | 50 (14.2) | 24 (16.0) | 41 (27.3) |
| Moderate dependence | 12 (3.4) | 6 (4.0) | 1 (0.7) |
| Severe dependence | 2 (0.6) | 3 (2.0) | 1 (0.7) |
| **Fear of disease progression**, n (%) |  |  |  |
| Absent | 263 (74.7) | 114 (76.0) | 113 (75.3) |
| Present | 89 (25.3) | 36 (24.0) | 37 (24.7) |

ACS: acute coronary syndrome; CCS: chronic coronary syndrome; IQR: interquartile range.

Supplementary Table 10. Standardized mean differences of predictors between the development and validation sets

| Variable | SMD (Development vs Internal Validation) | SMD (Development vs External Validation) |
| --- | --- | --- |
| **Gender** |  |  |
| Male | -0.027 | 0.100 |
| Female | 0.027 | -0.100 |
| **Educational level** |  |  |
| Junior high school | -0.038 | -0.208 |
| Senior high school/technical secondary school | -0.084 | 0.157 |
| College or above | -0.015 | 0.100 |
| Primary school or below | 0.122 | -0.033 |
| **Residence** |  |  |
| Urban | 0.005 | 0.087 |
| Rural | -0.005 | -0.087 |
| **Medical Coping Modes** |  |  |
| Confrontation | -0.010 | 0.057 |
| Avoidance | 0.005 | -0.178 |
| Resignation | -0.093 | -0.273 |
| **Exercise self-efficacy** | 0.030 | -0.061 |
| **Pain severity** |  |  |
| Mild pain | -0.118 | 0.062 |
| Moderate pain | 0.067 | -0.017 |
| Severe pain | 0.126 | -0.170 |
| **Clinical classification** |  |  |
| ACS | 0.193 | -0.461 |
| CCS | -0.193 | 0.461 |
| **Smoking status** |  |  |
| Never smoker | 0.080 | -0.015 |
| Former smoker | 0.017 | 0.001 |
| Current smoker | -0.097 | 0.014 |
| **Drinking status** |  |  |
| Never drinker | 0.072 | 0.032 |
| Former drinker | -0.126 | -0.159 |
| Current drinker | 0.041 | 0.116 |
| **Depression** |  |  |
| No depression | -0.138 | -0.151 |
| Possible depression | 0.125 | 0.042 |
| Depression | 0.044 | 0.147 |
| **Self-perceived burden** |  |  |
| No obvious burden | 0.019 | 0.086 |
| Mild burden | -0.074 | -0.032 |
| Moderate burden | 0.083 | -0.032 |
| Severe burden | -0.030 | -0.186 |
| **Barthel Index** |  |  |
| Independent | -0.095 | -0.249 |
| Mild dependence | 0.050 | 0.328 |
| Moderate dependence | 0.031 | -0.195 |
| Severe dependence | 0.127 | 0.013 |
| **Fear of disease progression** |  |  |
| Absent | 0.030 | 0.014 |
| Present | -0.030 | -0.014 |

ACS: acute coronary syndrome; CCS: chronic coronary syndrome; SMD: standardized mean difference; Absolute SMD values were reported to quantify differences in predictor distributions between the development and validation sets. An absolute SMD <0.10 indicates a negligible difference, 0.10–<0.20 a small difference, 0.20–<0.50 a moderate difference, and ≥0.50 a large difference.

Supplementary Table 11. Summary of predicted risk by observed outcome across study cohorts

| Cohort | Observed outcome | n | Predicted risk, Median (IQR) |
| --- | --- | --- | --- |
| Development set | Non-kinesiophobia | 127 | 0.188 (0.103–0.429) |
|  | Kinesiophobia | 225 | 0.890 (0.602–0.969) |
| Internal validation set | Non-kinesiophobia | 54 | 0.291 (0.165–0.569) |
|  | Kinesiophobia | 96 | 0.864 (0.574–0.944) |
| External validation set | Non-kinesiophobia | 62 | 0.144 (0.079–0.255) |
|  | Kinesiophobia | 88 | 0.934 (0.787–0.969) |

IQR: interquartile range.

Supplementary Table 12. Outcome prevalence across the development, internal validation, and external validation sets

| Cohort | Total, n | Kinesiophobia, n | Non-kinesiophobia, n | Prevalence (%) | *P* |
| --- | --- | --- | --- | --- | --- |
| Development set | 352 | 225 | 127 | 63.9 | 0.503 |
| Internal validation set | 150 | 96 | 54 | 64.0 |  |
| External validation set | 150 | 88 | 62 | 58.7 |  |

Supplementary Table 13. Ranking of Mean Absolute SHAP Values for the XGBoost Model

| Rank | Variable | Mean Absolute SHAP Value |
| --- | --- | --- |
| 1 | Resignation | 0.720 |
| 2 | Exercise self-efficacy | 0.635 |
| 3 | Avoidance | 0.380 |
| 4 | Fear of disease progression | 0.342 |
| 5 | Depression | 0.316 |
| 6 | Self-perceived burden | 0.309 |
| 7 | Confrontation | 0.156 |
| 8 | Gender | 0.141 |
| 9 | Smoking status | 0.135 |
| 10 | Residence | 0.094 |
| 11 | Educational level | 0.079 |
| 12 | Barthel Index | 0.039 |
| 13 | Clinical classification | 0.022 |
| 14 | Drinking status | 0.020 |
| 15 | Pain severity | 0.005 |

SHAP: SHapley Additive exPlanations.
